# A Novel View on the Taxonomy of Sulfate-Reducing Bacterium ‘*Desulfotomaculum salinum*’ and a Description of a New Species *Desulfofundulus salinus* sp. nov.

**DOI:** 10.3390/microorganisms12061115

**Published:** 2024-05-30

**Authors:** Tamara N. Nazina, Tatyana P. Tourova, Denis S. Grouzdev, Salimat K. Bidzhieva, Andrey B. Poltaraus

**Affiliations:** 1 Winogradsky Institute of Microbiology, Research Center of Biotechnology, Russian Academy of Sciences, 119071 Moscow, Russia; tptour@rambler.ru (T.P.T.); salima.bidjieva@gmail.com (S.K.B.); 2 SciBear OU, Tartu mnt 67/1-13b, 10115 Tallinn, Estonia; denisgrouzdev@gmail.com; 3 Engelhardt Institute of Molecular Biology, Russian Academy of Sciences, 119991 Moscow, Russia; abpolt@gmail.com

**Keywords:** *Desulfofundulus*, sulfate-reducing bacteria, genomics, spore-forming anaerobes, thermophiles, oil and gas reservoirs

## Abstract

Two thermophilic spore-forming sulfate-reducing strains, 435^T^ and 781, were isolated from oil and gas reservoirs in Western Siberia (Russia) about 50 years ago. Both strains were found to be neutrophilic, chemoorganotrophic, anaerobic bacteria, growing at 45–70 °C (optimum, 55–60 °C) and with 0–4.5% (*w*/*v*) NaCl (optimum, 0.5–1% NaCl). The major fatty acids were iso-C_15:0_, iso-C_17:0_, C_16:0_, and C_18:0_. In sulfate-reducing conditions, the strains utilized H_2_/CO_2_, formate, lactate, pyruvate, malate, fumarate, succinate, methanol, ethanol, propanol, butanol, butyrate, valerate, and palmitate. In 2005, based on phenotypic characteristics and a 16S rRNA gene sequence analysis, the strains were described as ‘*Desulfotomaculum salinum*’ sp. nov. However, this species was not validly published because the type strain was not deposited in two culture collections. In this study, a genomic analysis of strain 435^T^ was carried out to determine its taxonomic affiliation. The genome size of strain 435^T^ was 2.886 Mb with a 55.1% genomic G + C content. The average nucleotide identity and digital DNA–DNA hybridization values were highest between strain 435^T^ and members of the genus *Desulfofundulus*, 78.7–93.3% and 25.0–52.2%, respectively; these values were below the species delineation cut-offs (<95–96% and <70%). The cumulative phenotypic and phylogenetic data indicate that two strains represent a novel species within the genus *Desulfofundulus*, for which the name *Desulfofundulus salinus* sp. nov. is proposed. The type strain is 435^T^ (=VKM B-1492^T^ = DSM 23196^T^). A genome analysis of strain 435^T^ revealed the genes for dissimilatory sulfate reduction, autotrophic carbon fixation via the Wood–Ljungdahl pathway, hydrogen utilization, methanol and organic acids metabolism, and sporulation, which were confirmed by cultivation studies.

## 1. Introduction

O, be some other name!

What’s in a name! that which we call a rose

By any other name would smell as sweet…

*William Shakespeare, Romeo and Juliet*

For a long time, the genus *Desulfotomaculum* was the only known genus of spore-forming sulfate-reducing bacteria [1]. Application of marker genes and genomic approaches to a number of phenotypically similar spore-forming sulfidogens of the genus *Desulfotomaculum* led to the revision of this genus and establishment of new genera—*Desulfosporosinus*, *Desulfallas*, *Desulfofundulus*, *Desulfofarcimen*, and *Desulfohalotomaculum* [2,3].

Thermophilic sulfate-reducing spore-forming bacteria, strains 435^T^ (VKM B-1492^T^, DSM 23196^T^) and 781 (VKM B-1379), were isolated about 50 years ago from high-temperature hydrocarbon deposits in Western Siberia (Russia) [4]. The new isolates were phenotypically close to the species *Desulfotomaculum nigrificans* [1]. However, the strains had halotolerant properties, and it was proposed to classify them as a subspecies *Desulfotomaculum nigrificans* subsp. *salinus* [4]. Morphology, ultrastructural organization, and physiology, including the ability to fix nitrogen of strains 435^T^ and 781 were studied, and the results were reported in papers [5,6,7]. DNA-DNA hybridization and phylogenetic analysis of their 16S rRNA genes were performed, which made it possible to classify strains 435^T^ and 781 to the new species ‘*Desulfotomaculum salinum*’ and to propose a description of the species [8]. However, at this time, the type strain 435^T^ was deposited in only one collection of microorganisms (VKM B-1492^T^), and therefore the new species was not validly published. Later, strain 435^T^ was deposited in the German Collection of Microorganisms and Cell Cultures (DSMZ) under the number DSM 23196.

Sequencing and preliminary comparative analysis of the genome of strain 435^T^ [9] showed that it was most closely phylogenetically related to members of the genus *Desulfofundulus*. The overall genomic relatedness indices (OGRIs) of strain 435^T^, such as average nucleotide identity (ANI) and digital DNA-DNA hybridization (dDDH) were below the commonly used threshold of 95–96% and 70%, respectively, for species designation [10], and confirmed the independent species status of strain 435^T^ within the genus *Desulfofundulus*. The name ‘*Desulfofundulus salinum*’ has been proposed for the new species.

The genus *Desulfofundulus* was proposed by Watanabe et al. [3,11] based on a comparative phylogenetic analysis of the 16S rRNA genes of members of the polyphyletic genus *Desulfotomaculum*. The main features of the bacteria of the genus *Desulfofundulus* are rod-shaped cells, motility, spore formation, obligate anaerobiosis, thermophily, and the use of sulfate and thiosulfate as electron acceptors. *Desulfofundulus* spp. were isolated from subsurface thermal mineral water, formation water of oil reservoirs, hot springs, methanogenic reactors, and others. The genus *Desulfofundulus* belongs to the family *Peptococcaceae* of the order *Eubacteriales* in the class *Clostridia*, phylum *Bacillota*. Assignment of the genus *Desulfotomaculum* with the type species *Desulfotomaculum nigrificans* to the family *Desulfotomaculaceae* of the order *Eubacteriales* in the class *Clostridia* has recently been proposed [12].

At the time of writing this article in March 2024, the genus *Desulfofundulus* included nine species of thermophilic spore-forming sulfate-reducing bacteria—*D. kuznetsovii*, *D. australicus*, *D. luciae*, *D. solfataricus*, *D. thermoacetoxidans*, *D. thermobenzoicus*, *D. thermocisternus*, and *D. thermosubterraneus*; the genus also includes ‘*Desulfofundulus salinum*’ as a not validly published species (List of Prokaryotic Names with Standing in Nomenclature (LPSN), https://lpsn.dsmz.de/genus/desulfofundulus, accessed on 5 March 2024) [13]. The type species of the genus is *Desulfofundulus kuznetsovii* [3,14,15].

The purpose of this work was to combine previously published information on the phenotypic characteristics of thermophilic spore-forming sulfate-reducing bacterial strains 435^T^ and 781, to perform phylogenetic and genomic analyses, and to provide a formal description of the species *Desulfofundulus salinus* sp. nov.

## 2. Materials and Methods

### 2.1. Origin of Bacterial Strains

Strains 435^T^ (VKM B-1492^T^, DSM 23196^T^) and 781 (VKM B-1379) were isolated from samples of reservoir fluids taken by E.P. Rozanova from high-temperature hydrocarbon deposits (Western Siberia, Russia), as previously described [4]. The strain 435^T^ was isolated in 1973 by E.P. Rozanova from a mixture of condensation and reservoir water coming from well 435 of the Igrim gas field (63°06′2.12″ N, 64°19′54.15″ E), located in the Berezovsky district (Khanty-Mansiysk Autonomous Okrug, Yugra, Russia). The water came from terrigenous Jurassic sediments from a depth of 1600 to 1620 m. The reservoir temperature was 59 °C. The reservoir water belonged to the chloride–calcium type and contained 15.3 g of salt per liter. Strain 781 was isolated in 1974 by T.N. Nazina from a sample of reservoir water from production well 781 of the Ust-Balyk oil and gas field (61°05′ N, 72°18′33.33″ E), located in the Nefteyugansk district (Khanty-Mansiysk Autonomous Okrug, Yugra, Russia). The reservoir water came from the terrigenous strata of Cretaceous sediments from a depth of about 2100 m, belonged to the chloride–calcium type, and contained about 16.0 g of salts per liter. The reservoir temperature was 63–70 °C. Sulfates and hydrogen sulfide were absent in both studied water samples. Since the isolation, the strains have been stored in the Laboratory of Petroleum Microbiology, Winogradsky Institute of Microbiology, Research Center of Biotechnology, Russian Academy of Sciences.

### 2.2. Media Composition and Isolation

Postgate’s B, C, D, and N media were used to isolate the strains [16]; media were amended with 10–20% *v*/*v* formation water from the Ust-Balyk oil and gas field, which in subsequent experiments was replaced by 0.5–1.0% *w*/*v* NaCl. The strains were isolated by successive transfer from a liquid medium to a solid one and subsequent inoculation of single black colonies to a liquid medium. The strains were also cultivated in Widdel’s [17] medium brackish No. 1 containing the following (g/L distilled water): NaCl—5.0, MgCl_2_—0.6, KCl—0.3, CaCl_2_—0.1, NH_4_Cl—0.3, KH_2_PO_4_—0.2, Na_2_SO_4_—2.8, NaHCO_3_—1.5, and sodium lactate—3.5; the medium was reduced by Na_2_S·9H_2_O—0.3 and Na_2_S_2_O_4_—0.05. Trace elements [18] were added to the medium. The bacteria were cultured in Hungate’s tubes, and the gas phase was represented by molecular nitrogen or argon.

For comparative studies, *Desulfofundulus* (formerly *Desulfotomaculum*) *kuznetsovii* strain 17^T^ was used which, since its isolation, has also been stored in the collection of the Laboratory of Petroleum Microbiology, Winogradsky Institute of Microbiology, Research Center of Biotechnology, Russian Academy of Sciences [3,14,15]. Strain 17^T^ was cultured in Widdel’s medium for freshwater bacteria [17]. The strains were incubated at 60 °C unless otherwise indicated.

### 2.3. Phenotypic Characterization

The cell morphology was studied by phase contrast microscopy using a Zetopan light microscope (Reichert, Vienna, Austria). Gram staining of the cells was performed using a relevant kit (Merck KGaA, Darmstadt, Germany). The ultrastructural organization of the cells was studied in strains grown in Postgate’s B medium with pyruvate, as previously described [5]. Cells were examined under a transmission electron microscope JEM-100C (JEOL, Tokyo, Japan). The temperature, salinity, and pH ranges for the growth of strains 435^T^ and 781 were determined in a liquid Widdel’s medium with sulfate, in which fumarate (1 g/L) served as the substrate as described by Nazina et al. [8]. The range of substrates used was determined on media that did not contain yeast extract. Alcohols, malate, and oxalate were added in an amount of 1 g/L, and salts of other organic acids and sugars at 3 g/L. Possible electron acceptors for strains 435^T^ and 781 were determined on a Widdel’s medium with sodium lactate, in which the sulfate was replaced by thiosulfate (2.0 g/L), sulfite (1.0 g/L), elemental sulfur (1.5 g/L), or sodium nitrate (1 g/L). The possibility of thiosulfate dismutation was detected on a Widdel’s mineral medium, in which the sulfate was replaced with thiosulfate. The formation of SO_4_^2−^ was measured turbidimetrically [19]. The fixation of molecular nitrogen was determined by the acetylene method as the formation of ethylene, which was detected by gas chromatography [6]. The composition of cellular fatty acids was determined by gas chromatography–mass spectrometry on an HP-5973D device from Agillent Technologies (formerly Hewlett-Packard, Palo Alto, CA, USA) [8].

The growth of the strains was estimated by the optical density of the bacterial suspension at 660 nm, which was measured on an Ultrospec 2100 pro spectrophotometer (Amersham Biosciences, Amersham, Buckinghamshire, UK), as well as by the formation of sulfide, which was determined by the colorimetric method using N,N-dimethyl-paraphenylenediamine [20]. Growth experiments were performed in triplicate.

### 2.4. The 16S rRNA Gene and Genome Sequencing and Analysis

The methods for DNA isolation and sequencing of the 16S rRNA genes from strains 435^T^ and 781 were described earlier [8]. The 16S rRNA gene of strain 435^T^ was re-amplified using the strain’s template DNA and the universal bacterial primer set 27F (5′-AGAGTTTGATCCTGGCTCA-3′) and 1492R (5′-AGAGTTTGATCCTGGCTCAG-3′) [21]. DNA sequencing was performed on an ABI 3500 xL DNA analyzer automatic sequencer using an ABI PRISM^®^ BigDye™ Terminator v. 3.1 reagent kit (Thermo Fisher Scientific, Waltham, MA, USA) in accordance with the manufacturer’s recommendations.

The methods of isolation of genomic DNA of strain 435^T^, sequencing conditions, programs for checking the quality of the obtained sequences, and assembling scaffolds from contigs were described by Grouzdev et al. [9]. The average nucleotide identity (ANI) was determined using FastANI v. 1.3 [22]. Digital DNA-DNA hybridization (dDDH) of genomes was performed using the Genome-to-Genome Distance Calculator (GGDC) v. 2.1 [23]. The identification of protein-coding sequences and primary annotation of genes were performed using the NCBI Prokaryotic Genome Automatic Annotation Pipeline (PGAP) [24]. In addition, to identify protein-coding sequences, we used UniProt (https://www.uniprot.org/, release 2024_01, accessed on 24 January 2024) [25].

The 16S rRNA gene sequences were aligned using MUSCLE [26]. Phylogenomic analysis was performed using concatenated sequences of 120 marker genes obtained using GTDB-Tk version 1.0.2 [27]. The maximum likelihood tree of the 16S rRNA gene sequences was constructed with a GTR + F + I + G4 model, and the phylogenomic tree was constructed with a LG + F + I + G4 model recommended by ModelFinder [28] in IQ-TREE [29]. Branch supports were obtained with 10,000 ultrafast bootstraps [30]. Maximum parsimony and neighbor-joining trees were reconstructed using MPBoot [31] and MEGA11 [32], respectively. A pangenomic analysis was conducted using the bioinformatic pipeline proposed by Delmont and Eren [33], and Anvi’o version 7.0 [34]. Genomes were arranged based on the distribution of gene clusters using the MCL algorithm (Euclidean distance, Ward linkage).

The reconstruction of possible metabolic pathways was carried out based on comparison of the genome of the strain 435^T^ using BV-BRC (PATRIC) 3.35.5 (https://www.bv-brc.org/, accessed on 1 March 2024) [35], MetaCyc version 27.5 (https://metacyc.org/, accessed on 1 November 2023) [36], RAST v. 2.0 (https://rast.nmpdr.org/rast.cgi, accessed on 12 October 2023) [37] and the BlastKOALA tool, KEGG version 3.0 (https://www.kegg.jp/blastkoala/, accessed on 1 April 2023) [38]. Gene clusters of carbon dioxide fixation, sulfate reduction, propanoate degradation, and dinitrogen fixation were compared using an online service Gene Graphics version 2.02 (https://katlabs.cc/genegraphics/app, accessed on 1 October 2023) [39]. The circular genome map of strain 435^T^ was constructed using the Proksee web service version 6.0.3 (https://proksee.ca/, accessed on 5 March 2024) [40].

### 2.5. Nucleotide Sequence Accession Numbers

The GenBank/EMBL/DDBJ accession numbers for the 16S rRNA gene sequences of strains 435^T^ and 781 are AY918122 and AY918123, respectively. The genome shotgun project of strain 435^T^ was deposited at DDBJ/ENA/GenBank under accession no. RBWE00000000. The version described in this paper is the first version, RBWE01000000. The NCBI genomic assembly accession number of strain 435^T^ is GCF_003627965.1.

## 3. Results

In this study, the taxonomic position of sulfate-reducing bacterial strains 435^T^ and 781, previously described as ‘*Desulfotomaculum salinum*’ [8], was clarified; the genome of strain 435^T^ was analyzed and the indices of the genomic relationship were determined to clarify its taxonomic position, taking into account modern approaches. Genome analysis made it possible to correlate the genomic information and phenotypic features of strain 435^T^ and to give a formal description of the new species, *Desulfofundulus salinus* sp. nov.

### 3.1. Phylogenetic Analysis of the 16S rRNA Gene

**Figure 1:**
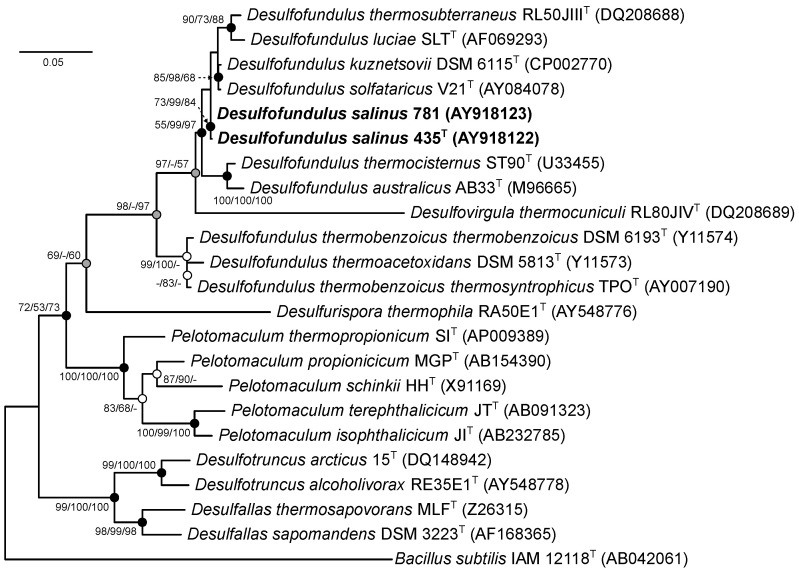
The maximum likelihood phylogenetic tree based on the 16S rRNA gene sequences (1369 nucleotide sites) reconstructed with the GTR + F + I + G4 evolutionary model, showing the position of strains 435^T^ and 781 within the genus *Desulfofunfulus*. Gray circles indicate that the corresponding nodes were recovered in the reconstructed tree based on the maximum parsimony algorithm; white circles indicate that the corresponding nodes were recovered using the neighbor-joining algorithm; black circles indicate that the corresponding nodes were also recovered based on the neighbor-joining and maximum parsimony algorithms. The bootstrap values (>50%) are listed as percentages at the branching points. Bar: 0.05 substitutions per nucleotide position. The tree was rooted using *Bacillus subtilis* IAM 12118^T^ as the outgroup. The GenBank accession numbers for 16S rRNA genes are indicated in brackets. The names of the strains described in this study are marked in boldface.

Phylogenetic analysis of 16S rRNA gene sequences in EzBioCloud and BLAST showed that strain 435^T^ showed a 99.4% similarity with strain 781 and a 98.4%, 98.0%, 97.5%, 95.6%, and 94.7% similarity with the respective genes of the nearest members of the genus *Desulfofundulus—D. solfataricus* V21^T^, *D. kuznetsovii* 17^T^, *D. thermocisternus* ST90^T^, *D. thermosubterraneus* RL50JIII^T^, and *D. luciae* SLT^T^. On the phylogenetic tree constructed using the NJ, MP, and ML methods, both strains formed a separate branch within the cluster of phylogenetically closely related *Desulfofundulus* species, including the type species of this genus *D. kuznetsovii* 17^T^, as well as *D. solfataricus* V21^T^, *D. luciae* SLT^T^, and *D. thermosubterraneus* RL50JIII^T^ (Figure 1).

This indicated that the studied strains 435^T^ and 781 belonged to the genus *Desulfofundulus*. Importantly, when the trees were constructed using the neighbor-joining and maximum likelihood algorithms, the genus *Desulfofundulus* appears to be polyphyletic. *D. thermoacetoxidans* and *D. thermobenzoicus* subsp. *thermosyntrophicus* formed a separate cluster on the phylogenetic tree, suggesting that further taxonomic revision may be required to fully resolve the evolutionary relationships within this group.

### 3.2. Genome Features and Phylogeny

The genome of the strain 435^T^ was composed of 10 scaffolds with a total genomic length of 2,886,683 bp, an N50 value of 121.7 kb, a G + C content of 55.1%, and coverage of 137× [9]. The genome comprised 2925 annotated genes, including 2827 protein-coding sequences, 42 pseudogenes, and 56 RNA genes. The ANI and dDDH values of the 435^T^ genome with the genomes of the type strains of the genus *Desulfofundulus* were in a range 78.7–93.3% and 25.0–52.2%, respectively (Table 1). These values were below the thresholds of 95–96% for ANI and 70% for dDDH, accepted for assigning bacteria to a single species [10,41], which indicates that strain 435^T^ belongs to a new species.

**Figure 2:**
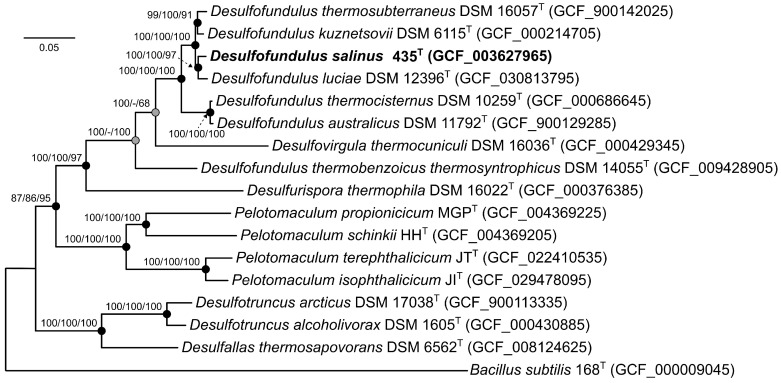
The maximum likelihood phylogenetic tree derived from concatenated 120 single-copy proteins showing the position of strain 435^T^ and closely related members within the genus *Desulfofundulus*. A phylogenetic analysis was performed with the LG + F + I + G4 model using 37,730 amino acid positions. Gray circles indicate that the corresponding nodes were recovered in the reconstructed tree based on the maximum parsimony algorithm; black circles indicate that the corresponding nodes were also recovered based on the neighbor-joining and maximum parsimony algorithms. Bar: 0.05 amino acid substitutions per site. The bootstrap values (>50%) are listed as percentages at the branching points. The tree was rooted using *Bacillus subtilis* 168^T^ as the outgroup. Accession numbers for the genomic assemblies are indicated in brackets.

To identify the phylogenetic position of strain 435^T^, a phylogenetic tree was constructed based on concatenated 120 single-copy proteins. On the phylogenetic tree, strain 435^T^ formed a separate branch in a cluster with the type strains *D. kuznetsovii* 17^T^, *D. luciae* SLT^T^, and *D. thermosubterraneus* RL50JIII^T^ (Figure 2).

As noted, a topology of the ribosomal tree (Figure 1), a low level of similarity of the 16S rRNA genes of *D. thermoacetoxidans* CAMZ^T^, *D. thermobenzoicus* subsp. *thermobenzoicus* TSB^T^, and *D. thermobenzoicus* subsp. *thermosyntrophicus* TPO with those of other members of the genus *Desulfofundulus*, as well as low values of the genomic relatedness indices dDDH, ANI, and a separate position on the phylogenomic tree of the strain *D. thermobenzoicus* subsp. *thermosyntrophicus* TPO, suggests that this phylogenetic group belongs to a separate new genus.

This assumption is also consistent with the GTDB database (release 08-RS214), which assigned *D. thermobenzoicus* subsp. *thermosyntrophicus* TPO to the distinct genus *Desulfofundulus*_A. However, for a reliable description of the new genus, information on the genomes of the type strains *D. thermoacetoxidans* CAMZ^T^ and *D. thermobenzoicus* subsp. *thermobenzoicus* TSB^T^ is required.

**Figure 3:**
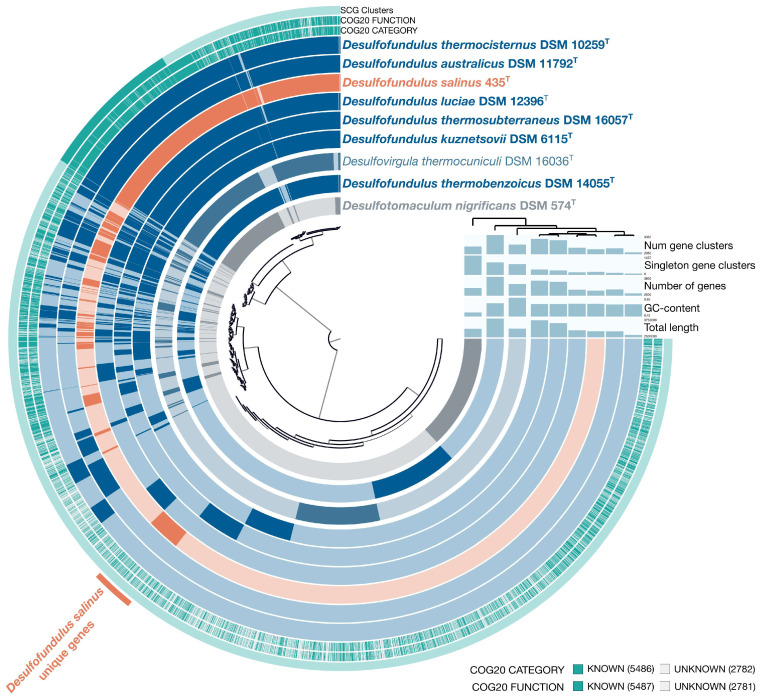
A pangenome analysis of type strains of *Desulfofunfulus* spp., *Desulfovirgula thermocuniculi*, and *Desulfotomaculum nigrificans* calculated with Anvi’o v. 7.0. The central dendrogram represents the relationships between 8268 gene clusters (28,350 genes) in the analyzed genomes. Dark circular regions represent the genes found in those areas for each genome. The phylogenetic tree was reconstructed using the single-copy genes.

A comprehensive pangenomic analysis was performed on genomes from seven *Desulfofundulus* strains and *Desulfovirgula thermocuniculi*, with the genome of *Desulfotomaculum* (*Dt.*) *nigrificans* as the outgroup. This dataset comprised 28,350 genes organized into 8268 gene clusters (GCs). Within this pangenomic landscape, 951 core clusters were consistently present across all genomes analyzed, of which 766 were single-copy genes (SCGs), as shown in Figure 3. The phylogenetic tree reconstructed from these SCGs, spanning 233,613 amino acid sites, mirrored the topology of the phylogenomic tree derived from concatenated analysis of 120 single-copy proteins. This tree confirmed the placement of strain 435^T^ within the genus *Desulfofundulus* and also indicated that *D. thermobenzoicus* subsp. *thermosyntrophicus* did not cluster with the type strains of *Desulfofundulus* spp., suggesting a distinct phylogenetic history.

The genome of strain 435^T^ possessed 224 GCs unique to other *Desulfofundulus* species, 66 of which had a function predicted according to the KEGG database This unique gene set underscores the specialized adaptations of strain 435^T^, particularly in adhesion, metabolic versatility, nutrient uptake, and defense mechanisms. Among its distinctive features, the genes encoding tight adhesion proteins B and C (K12510, K12511) and pilus assembly proteins (Flp/PilA K02651, CpaB K02279) suggest advanced capabilities for cellular adhesion and potentially for biofilm formation. Metabolic flexibility is indicated by the presence of the genes for 2-oxoglutarate ferredoxin oxidoreductase (K00177) and D-3-phosphoglycerate dehydrogenase (K00058), essential for carbon metabolism and serine biosynthesis.

The genome also harbors unique transport and membrane proteins, including tricarboxylic acid transport (K07793, K07794) and ABC transport system components (e.g., urea transport system ATP-binding protein K11962), coupled with sophisticated defense mechanisms against foreign DNA, as evidenced by the genes encoding components of type I restriction enzymes (K03427, K01154). Notably, the genome of strain 435^T^ contains the genes for urease activity (e.g., urease subunit alpha K01428, urease accessory protein K03188) and unique enzymes such as UDP-2-acetamido-2-deoxy-ribo-hexuluronate aminotransferase (K13017) and lactate racemase (K22373), highlighting its nitrogen utilization efficiency and essential enzymatic functions for cellular maintenance. A significant finding is the gene for long-chain acyl-CoA synthetase (EC: 6.2.1.3) (K01897), which is central to fatty acid metabolism, suggesting that strain 435^T^ has specialized lipid metabolic processes that differ from those of other *Desulfofundulus* species. This genomic characterization of strain 435^T^ enriches our understanding of its unique physiological capabilities and potential environmental adaptations, providing insights into metabolic diversity and evolutionary strategies within the genus *Desulfofundulus*.

### 3.3. Genome Insights

The analysis of enzyme composition of the metabolic pathways based on the results of functional protein prediction using the BlastKOALA and RASTtk services provides evidence that the genome of strain 435^T^ contains all the key genes responsible for complete pathways of carbohydrate metabolism: glycolysis (the Embden–Meyerhof pathway) and glucogenesis, pentose phosphate pathway, UDP-N-acetyl-D-glucosamine biosynthesis, and propionate oxidation. Complete sets of the genes comprising the reductive Acetyl-CoA pathway for CO_2_ fixation (the Wood–Ljundahl pathway) were revealed, as well as those of the pathways for dinitrogen fixation, biosynthesis of the major amino acids, dissimilatory sulfate reduction, biosynthesis and beta-oxidation of fatty acids, dTDP-L-rhamnose biosynthesis, and biosynthesis of vitamins and cofactors as follows: NAD, FAD, biotin (BioW pathway), L-valine, and coenzyme A. No genes of complete pathways for xenobiotic degradation were found. The circular genome map (Figure S1) of strain 435^T^ shows a localization of the genes presumably involved in the metabolic pathways, according to the genome analysis carried out in this work.

#### 3.3.1. Autotrophic CO_2_ Fixation

Genomic analysis confirmed the previously shown ability of strain 435^T^ to grow on molecular hydrogen and carbon dioxide (or formate) in the presence of sulfate as an electron acceptor. According to the KEGG map ’Carbon fixation pathways in prokaryotes’ (id. 00720), in the genome of strain 435^T^, all genes are annotated of the complete reductive acetyl-CoA pathway (the Wood–Ljungdahl pathway), including the genes of key enzymes, anaerobic carbon-monoxide dehydrogenase catalytic subunit (EC: 1.2.7.4) and acetyl-CoA synthase (EC: 2.3.1.169) (Figure S2). This pathway is used by many anaerobic bacteria, but the structure of the gene clusters encoding enzymes of this pathway differs from taxa to taxa. Examples of such different structures are given by Pierce et al. [42] in comparison with the annotated structure in the genome of the type strain *Moorella thermoacetica* ATCC 39073^T^.

In the genome of strain 435^T^, the genes of this pathway are present as the carbonyl branch operon, starting with the *cooC* gene (D7024_09170) encoding the CO dehydrogenase maturation factor, and the next cluster of genes of the CO complex *acsA(cooS)BCDE* (D7024_09175-09200) encoding the main and auxiliary genes of dehydrogenase/acetyl-CoA synthase (EC: 1.2.74/2.3.1.169). The structure of this operon is identical for the genomes of the type strains 435^T^, *D. kuznetsovii* 17^T^, and *Desulfoscipio* (*Ds.*) *gibsoniae* DSM 7213^T^; however, in the genomes of *Desulfofarcimen* (*Df.*) *acetoxidans* DSM 771^T^ and *M. thermoacetica* ATCC 39073^T^, this operon differs by the insertion of the ferredoxin gene ahead of the second *cooC* gene (Figure S3).

**Figure 4:**
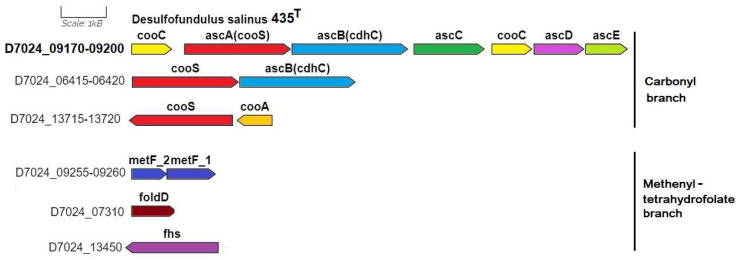
The genes presumably encoding the enzymes of the Wood–Ljungdahl pathway in the genome of the *D. salinus* strain 435^T^. Abbreviations: *acsA (cooS),* carbon monoxide dehydrogenase; *acsB*, acetyl-CoA synthase; *ascC*, acetyl-CoA synthase corrinoid iron-sulfur protein, large subunit; *ascD*, acetyl-CoA synthase corrinoid iron-sulfur protein, small subunit; *acsE*, methyltetrahydrofolate methyltransferase; *cooC*, carbon monoxide dehydrogenase maturation factor; *metF_1-2*, methylenetetrahydrofolate reductase; *foldD*, methenyltetrahydrofolate cyclohydrolase/methylenetetrahydrofolate dehydrogenase (NADP+); and *fhs*, formate-tetrahydrofolate ligase. Scale bar, 1000 bp.

The genes encoding the methenyltetrahydrofolate branch in the genome of strain 435^T^ are located separately. They include *metF1-2* (D7024_9255-09260), encoding the enzyme methylenetetrahydrofolate reductase (EC: 1.5.1.54), *foldD* (D7024_07310), encoding a bifunctional enzyme methenyl-tetrahydrofolate cyclohydrolase (EC: 3.5.4.9)/methylenetetrahydrofolate dehydrogenase (NADP+) (EC: 1.5.1.5), and *fhs* (D7024_13450), encoding formate-tetrahydrofolate ligase (EC: 6.3.4.3). Interestingly, a duplicate of the main carbonyl branch *ascA(cooS)B* (D7024_6415-06420) genes is additionally annotated in the genome of strain 435^T^. The *cooS* gene is annotated in a cluster with the *cooA* (D7024_13715-13720) gene, which determines the carbon monoxide-responsive transcriptional activator. The localization of all genes encoding enzymes of the reductive acetyl-CoA pathway in the genome of strain 435^T^ is shown in (Figure 4).

Although the growth of strain 435^T^ on CO has not been experimentally tested, the presence of several *cooS* genes encoding CO hydrogenase in their genome allows us to assume this possibility. The ability to use CO was also observed in a number of other sulfate-reducing bacteria, in particular, in a type strain of *Desulfotomaculum nigrificans* ATCC 19998^T^, as well as in another strain of this species, CO-1-SRB, which was previously assigned to a separate species due to its ability to grow on CO [43,44].

The genes *fdhAB* (d7024_02675_02680) determining the enzyme confurcating selenocysteine-incorporated formate dehydrogenase (NADP^+^) (EC: 1.17.1.10) are also annotated in the genome of strain 435^T^. This enzyme catalyzes both the fixation of CO_2_ via formate and the reverse oxidation reaction of formate to carbon dioxide, which corresponds to the revealed ability of this strain to grow on formate.

The ability to oxidize acetate to CO_2_ via the reverse acetyl-CoA pathway has been shown for some sulfate reducers, including *Df. acetoxidans* DSM 771^T^, based on the isolation of all the relative enzymes and determination of their activity [45,46]. The genome of *Df. acetoxidans* DSM 771^T^ was subsequently found to contain all the genes required for this process [47]. A similar ability was suggested for *D. kuznetsovii* type strain 17^T^, which is related to strain 435^T^ and grows on acetate in the presence of sulfate [14]. The acetyl-CoA pathway gene clusters from genomes of these strains had a similar structure; however, unlike *Df. acetoxidans* DSM 771^T^ and *D. kuznetsovii* 17^T^, the initial study did not report acetate consumption by strain 435^T^ [4]. Belyakova and Rozanova subsequently observed very slow acetate consumption during long-term incubation (56 days) [7]. No acetate consumption was also shown for another sulfate reducer, *Ds. gibsoniae* DSM 7213^T^, in the genome of which a similar one was annotated [48]. Four *asc* genes, coding another pathway of acetate oxidation via acetyl-CoA synthetase (EC: 6.2.1.1), were annotated in the genome of strain 435^T^: D7024_03470, 06805, 10610, and 10675. These genes are also present in the genomes of *D. kuznetsovii* 17^T^ and *Ds. gibsoniae* DSM 7213^T^, unlike the genome of *Df. acetoxidans* DSM 771^T^, in which the genes of other enzymes for acetate oxidation were annotated: *ackA* encoding acetate kinase (EC: 2.7.2.1) and *pta* encoding phosphate acetyltransferase (EC: 2.3.1.8). Although it is presently still unclear why sulfate reducers with similar genetic structures for acetate oxidation differ in their ability to grow on this substrate, the detected genes determining acetate consumption enzymes may probably make strain 435^T^ capable of complete oxidation of organic compounds.

#### 3.3.2. Sulfur Metabolism

**Figure 5:**
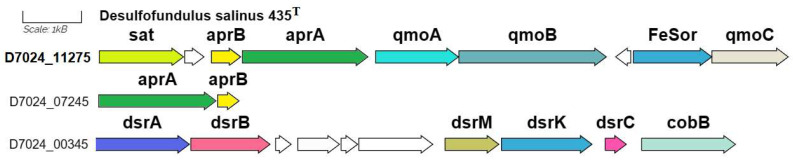
The genes presumably encoding the process of dissimilatory sulfate reduction in the genome of *D. salinus* strain 435^T^. Abbreviations: *sat*, sulfate adenylyltransferase; *aprAB*, adenylylsulfate reductase; *qmoABC*, proteins of adenylsulfate reductase-associated electron transfer complex; *dsrABD*, dissimilatory sulfite reductase; *dsrMK*, [DsrC]-trisulfide reductase; *dsrC*, sulfur redox associated protein; and *cobB*, protein similar to cobyrinic acid a,c-diamide synthetase clustered with dissimilatory sulfite reductase. Scale bar, 1000 bp.

According to the KEGG map of sulfur metabolism, strain 435^T^ reduces sulfate only via the dissimilatory process (Figure S4). The genes of dissimilatory sulfate reduction to sulfite are represented in the genome of strain 435^T^ by two clusters, one of which comprises the *sat* gene (D7024_11275) encoding sulfate adenylyltransferase (EC: 2.7.7.4), the genes *aprAB* (D7024_07245-07250) encoding two subunits of adenylylsulfate reductase (EC: 1.8.99.2), and the *qmoABC* genes encoding the proteins of adenylsulfate reductase-associated electron transfer complex (Figure 5). Another set of the *aprAB* genes (11260-11265) is represented by a separate cluster. Further sulfate reduction to sulfide via the intermediary metabolite [DsrC-protein]-trisulfide is carried out by dissimilatory sulfite reductase (EC: 1.8.1.22), two subunits of which are determined by the *dsrABD* cluster (D7024_00345-00355) and by [DsrC]-trisulfide reductase (EC: 1.8.5.10). The latter is coded by a truncated cluster of genes of the *dsrMK* (D7024_00375-00380) and by the associated *dsrC* dissimilatory complex (D7024_00375-00385) encoding sulfur redox associated protein. This localization of the genes of dissimilatory sulfate reduction is identical to the orthological one in the genomes of other *Desulfofundulus* strains, including *D. kuznetsovii* 17^T^.

The KEGG map of sulfur metabolism (Figure S4) also presents the enzyme thiosulfate reductase/polysulfide reductase (EC: 1.8.5.5), which is tentatively involved in thiosulfate disproportionation to sulfate and sulfide. This process has been studied mainly in Gram-negative bacteria, including sulfate-reducing *Deltaproteobacteria*, although it was hypothesized to be common to all sulfate reducers [49]. While the ability to grow by thiosulfate disproportionation with the formation of stoichiometric amounts of sulfate and sulfide has been shown for the *D. thermobenzoicus* type strain, this ability was not revealed for the type strains of *Dt*. *nigrificans*, *Dt*. *ruminis*, and *Desulfosporosinus orientis* [50]. The genomes of *D. thermobenzoicus* subsp. *thermosyntrophicus* strains TPO and 435^T^ (D7024_03870) were found to possess the homologous genes (80% identity of translated amino acid sequences), which were annotated by the BlastKOALA service as the *phsA* (*psrA*) genes coding the enzyme thiosulfate reductase/polysulfide reductase (EC: 1.8.5.5), which probably indicated their functional identity. This was in agreement with the previously shown ability of strain 435^T^ to carry out thiosulfate dismutation to sulfate and sulfide [8].

Among the genes encoding the enzymes of assimilatory sulfate reduction, the genome of strain 435^T^, similar to that of *D. kuznetsovii* 17^T^, possessed only *cysH* (D7024_00280), coding phosphoadenosine phosphosulfate reductase (EC: 1.8.1.8).

#### 3.3.3. Carbohydrate Metabolism and Oxidation of Organic Compounds

Strain 435^T^, as well as *D. kuznetsovii* 17^T^, was unable to grow on glucose or fructose as electron sources in the presence of sulfate [8]. However, the genomes of both strains contained all the genes encoding the enzymes involved in the Embden–Meyerhof–Parnas’s pathway, as well as in the pentose phosphate pathway, a non-oxidative phase. At the same time, the genes of the main enzymes of the Entner–Doudoroff pathway were not detected in the genomes of both strains, as well as most of the genes encoding the enzymes of the Krebs cycle.

Strain 435^T^ oxidizes organic substrates such as lactate, pyruvate, malate, fumarate, and succinate in the presence of sulfates. The genome of strain 435^T^ contains the *ldh* (D7024_03275) gene encoding L-lactate dehydrogenase (EC: 1.1.1.27), which is involved in the oxidation of lactate to pyruvate. The genome of strain 435^T^ also comprises the genes *maeA* (D7024_09930), *fumAB* (D7024_09975-09980), and *frdABC* (D7024_12330-12340), coding malate dehydrogenase (EC: 1.1.1.38), fumarate hydratase (EC: 4.2.1.2), and succinate dehydrogenase (EC: 1.3.5.1), respectively, which may be involved in the oxidation of malate, fumarate, and succinate to pyruvate. Based on the similarity of the gene clusters (Figure S5) encoding the enzymes of propionate oxidation (Figure S6) with those previously presented for *D. kuznetsovii* 17^T^ [51], this metabolic process in strain 435^T^ can proceed in a similar way. Strain 435^T^ can use the enzyme pyruvate formate-lyase (EC: 2.3.1.54) encoded by the *pflD* (D7024_11580) gene to ferment pyruvate to acetyl-CoA.

#### 3.3.4. Methanol Metabolism

Methanol may be consumed via the cobalt-dependent methyl transferase (MT) pathway in combination with the reductive acetyl-CoA pathway, as has been tentatively described for the *M. thermoacetica* type strain ATCC 39073^T^ [42]. The possibility of the MT pathway for methanol degradation to CO_2_ was confirmed for *D. kuznetsovii* 17^T^ by proteomic analysis under conditions of growth in the presence of cobalt and vitamin B12 [52]. Key genes of the MT pathway were annotated in the genome of strain 435^T^: *mtaA* (D7024_14080, 14030) [methyl-Co(III) methanol-specific corrinoid protein]:coenzyme M methyltransferase (EC: 2.1.1.246), *mtaB* (D7024_14075) methanol-corrinoid protein Co-methyltransferase (EC: 2.1.1.90), and *mtaC* (D7024_14070) methanol methyltransferase corrinoid protein, which, similar to the case of *D. kuznetsovii* 17^T^, are components of the complete MT operon. A comparison of the structures of these MT operons (Figure S7) revealed 94% homology of their nucleotide sequences, which indicated the possible existence of the MT pathway for methanol consumption in strain 435^T^. At the same time, the complete MT operon was not revealed in the genome of another *D. kuznetsovii* strain, TROSR, and proteomic analysis did not confirm the ability of strain TROSR to carry out methanol degradation via the MT pathway [53].

Methanol-oxidizing activity has also been shown for the partially purified alcohol dehydrogenase (ADH) (EC: 1.1.1.1) with a molecular mass of ~42 kDa from the type strain *D. kuznetsovii* 17^T^ [54]. The genome of this strain contained several genes determining ADH with a similar mass, which suggested the involvement of these enzymes in methanol assimilation [51]. A proteomic analysis revealed [52] that during growth on methanol in the absence of cobalt in the medium, the activity of the methyl transferase system decreased significantly, while the activity of one of the ADHs increased (Desku_2952), together with that of aldehyde ferredoxin oxidoreductase (Desku_2951), which confirmed the possibility of the existence of another pathway for methanol assimilation via the ADH pathway. Methanol is probably oxidized by ADH to formaldehyde, which is then oxidized to the formate by an aldehyde ferredoxin oxidoreductase. The genome of strain 435^T^ was also found to contain several ADH-determining genes (D7024_02205, 2710, 2725, 11765, 11800, 11860, and 13790). The translated amino acid sequence of one of them, D7024_02725, has a 99% identity with the sequence of the gene of the tentatively active ADH from *D. kuznetsovii* 17^T^ (Desku_2952). This may indicate the possible functioning of a cobalt-independent, NAD-dependent alcohol dehydrogenase (ADH) pathway of methanol oxidation in strain 435^T^.

Unlike *D. kuznetsovii* 17^T^, in the genome of strain 435^T^ the *mdh* gene (D7024_11860) was annotated, which encodes methanol dehydrogenase (EC: 1.1.1.244), also oxidizing methanol to formaldehyde. The genes of specific enzymes of formaldehyde assimilation were, however, not annotated (Figure S8). In this strain, methanol dehydrogenase is probably also involved in methanol assimilation via formaldehyde, together with aldehyde ferredoxin oxidoreductase. Further research is required to elucidate the contribution of different metabolic pathways to the processes of methanol degradation by strain 435^T^.

#### 3.3.5. Methane Metabolism

The formation of small amounts of so-called mini-methane has been previously reported for strains 435^T^ and 781 grown under lithoautotrophic conditions on H_2_/CO_2_ [7]. However, the KEGG map did not reveal complete modules of the known types of methanogenesis, including hydrogenotrophic one from H_2_/CO_2_, since the genome of strain 435^T^ does not encode the enzyme of the terminal stage of methanogenesis, methyl-coenzyme M reductase (EC: 2.8.4.1), which catalyzes the conversion of methyl-CoM to methane (Figure S8). The previously obtained results were probably caused by an experimental error.

#### 3.3.6. Nitrogen Metabolism

The ability of strain 435^T^ to fix molecular nitrogen, albeit at a very low rate, which has been shown by an acetylene test [6], was analyzed at the genome level. According to the KEGG map of nitrogen metabolism, nitrogenase (EC: 1.18.6.1) is present among its enzymes (Figure S9). The genome of strain 435^T^ contains the nitrogenase reductase *nifH* gene in the Nif cluster (D7024_03585-D7024_03620), which also comprises the genes of nitrogenase subunits *nifD* and *nifK*, the *nifB* and *NifE* auxiliary genes, and the genes determining the P-II-family proteins of nitrogen metabolism regulators (Figure S10). The Nif cluster was flanked by the *nrgA* gene, determining an ammonium transporter protein, and the *nifV* gene encoding homocitrate synthase. A similar gene cluster was annotated in most genomes of the type strains of *Desulfofundulus* species, including *D*. *kuznetsovii* DSM 6115^T^ (=17^T^). However, according to the previously obtained physiological data, this strain was completely incapable of nitrogen fixation [14]; for other *Desulfofundulus* strains, this ability has not been studied.

The reason for the absence or very low level of nitrogen-fixing ability in the studied *Desulfofundulus* strains, in spite of the presence of the Nif clusters in their genomes, is still unclear. A partially similar nitrogenase gene cluster was annotated in the genomes of *Desulfomicrobium baculatum* X^T^ [55] and *Desulforamulus ruminis* DSM 2154^T^, for which active dinitrogen fixation was shown by the acetylene method [6,56]. However, apart from the Nif gene clusters, similar to those of strain 435^T^ and other *Desulfofundulus* strains, the genomes of *Desulfomicrobium baculatum* X^T^ and *Desulforamulus ruminis* DSM 2154^T^ were also found to contain the *nifN* genes, which some authors consider essential in the minimal set of auxiliary proteins for nitrogenase maturation [57]. The absence of these genes may be responsible for the very low nitrogenase activity of strain 435^T^ and for the complete absence of this activity in *D. kusnetsovii* 17^T^. Another cause may be the complete or partial inactivation of some nitrogenase genes, resulting, for instance, from fragmentation, as has been revealed for the *NifD* alpha-subunit genes in the genomes of some *Desulfofundulus* strains. It may be noted that *Dt. nigrificans* DSM 574^T^ also lacks the ability to fix dinitrogen, in spite of the presence of both auxiliary genes, *nifE* and *nifN*, in its Nif cluster [56], probably due to fragmentation of the *nifK* nitrogenase beta-subunit gene (Figure S10). Further research on nitrogen fixation in Gram-positive sulfate reducers at the physiological and genomic levels is therefore required.

No enzymes for assimilatory and dissimilatory nitrate reduction were annotated in the genome of strain 435^T^, which was in agreement with the results of physiological tests, showing that strain 435^T^ did not use nitrate as an electron acceptor for growth [8]. According to the results of the KEGG analysis of the nitrogen metabolism pathways (Figure S9), the genes *gudB*, *glnA*, and *gltB* were annotated, which determine the enzymes for assimilation of ammonium, probably produced via nitrogen fixation, namely, NAD-specific glutamate dehydrogenase (EC: 1.4.1.2), glutamine synthetase (EC: 6.3.1.2), and glutamate synthase (EC: 1.4.1.13), respectively.

#### 3.3.7. Hydrogenase Genes

The *hydABC* genes (D7024_02610-2620) encoding bifurcating trimeric [FeFe] hydrogenase (EC: 1.12.1.4) are present in the genome of strain 435^T^. This gene cluster is associated with the gene (D7024_02625), which determines the PAS domain-containing protein, tentatively involved in the regulation of the synthesis of this hydrogenase [58]. This hydrogenase was originally found in *Thermotoga maritima* DSM 3109 [59]. According to the MetaCyc data, it is responsible for the irreversible metabolic pathway hydrogen production I. This heterotrimeric enzyme catalyzes the NADH- and ferredoxin-dependent proton reduction to H_2_ by a single enzyme complex; it functions only under anoxic conditions. The annotated gene cluster *hydABC* (D7024_02610-02625), determining trimeric confurcating [FeFe]-hydrogenases, was homologous to one of two clusters of the hydrogenase genes of the *D. kuznetsovii* 17^T^ genome [51]. In the genome of strain 435^T^, the genes of the *hndABCD* operon (D7024_05825-5835, 5815), determining tetraheteromeric NADP-dependent [Fe-Fe] hydrogenase (EC: 1.12.1.3), were annotated. This hydrogenase has been thoroughly studied for the sulfate reducer *Solidesulfovibrio fructosivorans* DSM 3604 [60]. According to the MetaCyc data, this hydrogenase carries out hydrogen oxidation under anoxic conditions, using reduced sulfur compounds as the terminal electron acceptors. In the absence of these compounds, the reverse process is possible, producing hydrogen during fermentative growth on relevant carbon sources (e.g., pyruvate for strain 435^T^). The genes *hygEFG* (D7024_05780-05790), coding the proteins facilitating the maturation of [Fe-Fe] hydrogenase [61], were annotated at the same genome site.

#### 3.3.8. Sporulation Genes

It was previously shown that in the genus *Desulfotomaculum,* the initial (zero) stage of sporulation is similar to the relevant stage in *Bacillus*, rather than in *Clostridium* [62]. It is initiated by the autophosphorylation of a sensory histidine kinase, followed by the sequential transfer of the phosphoryl group to the Spo0F and SP0B proteins, and culminates in the accumulation of Spo0A∼P, the main regulator of sporulation. In the genomes of members of the genus *Desulfotomaculum*, including *D. kuznetsovii* 17^T^, which has previously been assigned to this genus, conserved genetic structures homologous to those of bacilli were identified, including the *spo0B* gene encoding a sporulation response regulatory protein, as well as *rplU,* encoding for a ribosomal protein L21, *rpmA* for a ribosomal protein L27, and *obgE* for an Obg family GTPase. The genome of strain 435^T^ also has such a structure (D7024_12390-12375), so it can be assumed that the D7024_12385 gene is a homolog of the *spo0B* gene of *D. kuznetsovii* 17^T^. According to the results of the BlastKOALA service, other genes of sporulation stage zero are also annotated in the genome of strain 435^T^, including *spo0F* (D7024_00550, 03180) and *spo0A* (D7024_07375), as well as 48 *spo* genes of subsequent I–V stages of sporulation and 10 genes of the sensory histidine kinase, which corresponds to the revealed ability of this strain to complete sporulation [5,8].

### 3.4. Phenotypic Characteristics of Strains 435^T^ and 781

**Figure 6:**
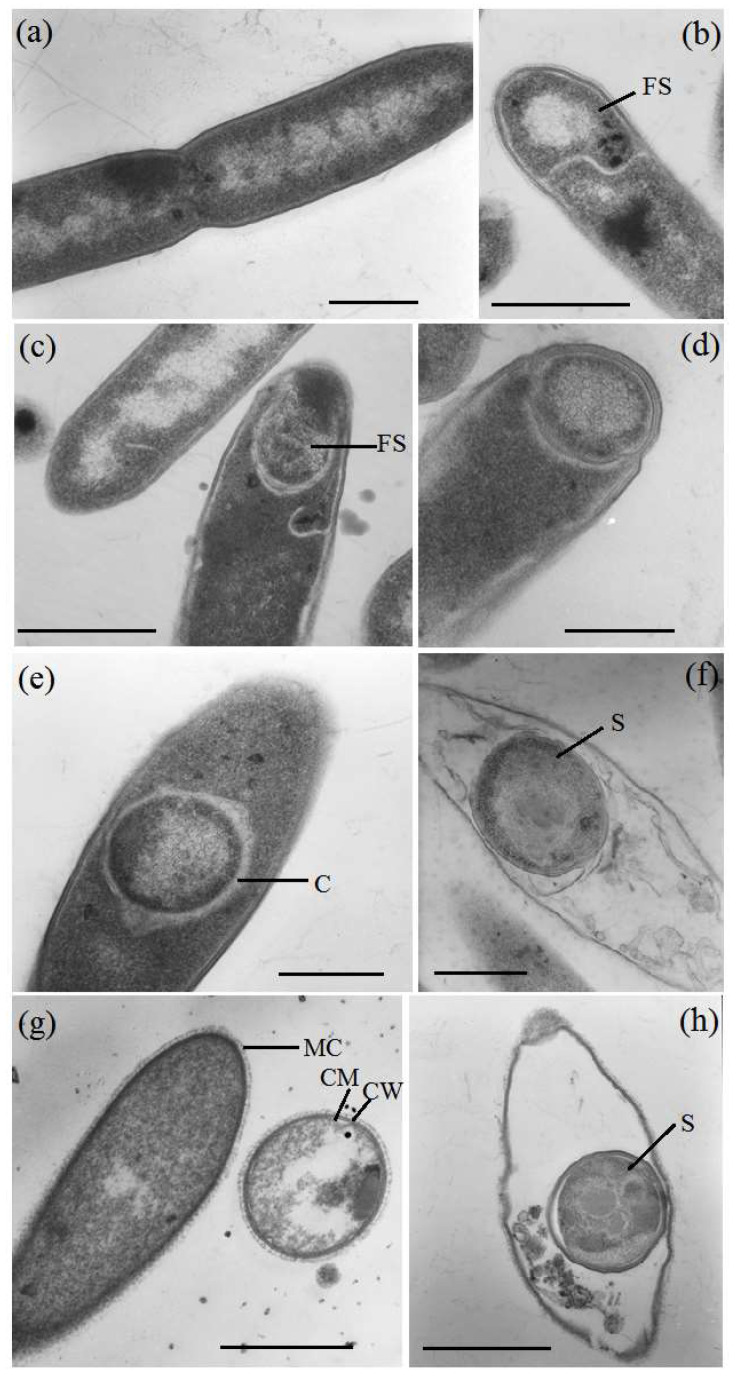
Transmission electron photomicrographs of thin sections of cell division in strain 781 during vegetative growth (**a**), sporulation (**b**–**f**), ultrastructure of strain 435^T^ cells (**g**), and spores (**h**). Designations: CM, cytoplasmic membrane; CW, cell wall; C, cortex; MC, microcapsule; FS, forespore; and S, spore. Bar (**a**,**d**,**e**,**f**), 0.5 µm; bar (**b**,**c**,**g**,**h**), 1.0 µm.

Cells of strains 435^T^ and 781 were rod-shaped or spindle-shaped (lemon-shaped), with sizes of 0.9–1.3 × 2–5 µm and 0.6–1.0 × 2–5 µm, respectively. The cells were motile due to peritrichous flagella, which are spore-forming. Despite the fact that the strains belong to the group of Gram-positive bacteria of the phylum *Bacillota*, they were Gram-negative stained, which was also noted for other members of this genus [53]. However, electron microscopic examination of the cells showed a cell wall structure typical of Gram-positive bacteria (Figure 6) [8]. The cell wall lacked the external lipoprotein membrane characteristic of Gram-negative bacteria.

Membrane structures that are invaginations of the cytoplasmic membrane, the nucleoid, and polyribosomes were visible in the cytoplasm. It has been shown that cell division is carried out by forming a septum in the central part of the cell, producing two daughter cells of equal sizes (Figure 6a). After division, the cells separated, although chains of two and four cells occurred in the culture. The strains formed spores of oval or spherical shape, located in the center of the cell or subterminal, and slight swelling of the cells. Figure 6b–f show spores at different stages of maturation, spore coat and a cortex, located between the inner and outer membranes of the forespore. Similar to *Bacillus* species [63,64], during sporulation, the strains 435^T^ and 781 formed a division septum located closer to one pole, producing a smaller forespore and a larger mother cell (Figure 6b–d). Subsequently, the forespore was engulfed by the mother cell, producing an internal endospore that after maturation is released by mother cell lysis (Figure 6e,f).

The main differentiating features of strains 435^T^ and 781 and members of the genus *Desulfofundulus* are shown in Table 2. Both strains were adapted to the temperature and salinity of the habitats from which they were isolated.

The optimum temperature for the growth of strains 435^T^ and 781 was 55–60 °C, with weak growth observed at 40–45 °C and 65–70 °C, respectively (Figure S11a). Strains 435^T^ and 781 grew in the salinity ranges 0–3 and 0–4.5% NaCl (*w*/*v*), respectively, with optima at 1% and 0.5% NaCl (Figure S11b). The strains remained viable after cultivation in a medium with 6% NaCl, probably due to the formation of spores, but no increase in biomass was observed. Both strains were neutrophils, growing in the pH range of 6.0–8.5; the strains accumulated the maximum amount of biomass at pH 7.0, whereas the maximum formation of sulfide in the medium was noted at pH 8.5 [8].

Both strains were obligate anaerobes and had a similar range of electron donors and acceptors used. The strains reduced sulfate to hydrogen sulfide in media with lactate, pyruvate, formate, propionate, butyrate, valerate, palmitate, malate, fumarate, succinate, methanol, ethanol, propanol, and butanol and were capable of autotrophic growth in a mineral medium with a H_2_/CO_2_ gas phase. L-alanine, L-serine, L-arginine, asparagine, L-cysteine, glucose, fructose, lactose, benzoate, citrate, tartrate, glycerol, glutamate, threonine, tryptophan, and phenylalanine were not used. The strains carried out an incomplete oxidation of lactate and other organic substrates to acetate. In our experiments (up to 14–30 days), growth on acetate coupled with sulfide production was not observed. However, very slow acetate consumption by both strains after 56 days of cultivation has been noted [7].

The strains 435^T^ and 781 used sulfate, sulfite, and thiosulfate, but not nitrate as electron acceptors and carried out thiosulfate dismutation to sulfate and sulfide. In the absence of sulfate, the bacteria fermented pyruvate with acetate production, and weak growth was also observed on fumarate; lactate was not fermented. The cellular fatty acid profiles of strains 435^T^, 781, and *D. kuznetsovii* 17^T^ were dominated by the same fatty acids that were found earlier [8], but in other quantitative ratios. The major fatty acids of strains 435^T^ and 781 were *iso*-C_15:0_ (22.1 and 32.7% of the total amount of fatty acids, respectively), *iso*-C_17:0_ (9.4 and 18.0%), C_16:0_ (21.1 and 25.5%), and C_18:0_ (31.5 and 10.7%) fatty acids (Table S1). In the fatty acids profiles of the type strains of *D. luciae* [69], *D. solfataricus* [68], and *D. thermosubterraneus* [69], *iso*-C_15:0_ and *iso*-C_17:0_ fatty acids were also predominant. Desulfoviridin was not detected.

The strains 435^T^ and 781 can be distinguished from the closest members of the genus *Desulfofundulus* by their phenotypic characteristics, including a range of utilized substrates (Table 2). The data presented show that physiological features of strain 435^T^ were supported by the metabolic pathways predicted from its genome sequence. The results of phenotypic studies, phylogenetic position based on the 16S rRNA gene sequencing, and genomic analysis allow us to assign the strains 435^T^ and 781 to a new species of the genus *Desulfofundulus* with the proposed name *Desulfofundulus salinus* sp. nov. The description of the species is given in the protologue (Table 3).

The ability to grow autotrophically on H_2_/CO_2_, as well as to use lower alcohols and fatty acids, together with the optima of temperature and salinity for strains 435^T^ and 781, indicate the adaptation of the strains to the conditions of the deep subsurface horizons from which they were isolated. A strain distantly related to ‘*Desulfotomaculum salinum*’ was isolated from the sulfate-reducing culture obtained from a black rust deposit from a borehole on the eastern flank of Juan de Fuca Ridge [73]. A phylotype of ‘*Desulfotomaculum salinum’* (99% of 16S rRNA gene sequence similarity) was detected by the DGGE molecular approach in water from a borehole located on the west bank of the river Ob’ (58°50′ N, 81°30′ E) in the Tomsk Region, Western Siberia, Russia [74]. Thus, thermophilic spore-forming sulfate-reducing bacteria of the *Desulfotomaculum*–*Desulfofundulus* group are commonly found inhabitants of deep subsurface environments [75].

## 4. Conclusions

In this study, two thermophilic spore-forming sulfate-reducing strains, 435^T^ and 781, isolated 50 years ago from hydrocarbon deposits of Western Siberia (Russia), were taxonomically characterized using physiological, chemotaxonomic, and phylogenetic approaches. Based on phenotypic and 16S rRNA gene-based analyses, the strains were previously assigned to the genus *Desulfotomaculum*, as ‘*Desulfotomaculum salinum*’. A genome analysis of the type strain 435^T^ made it possible to bring the taxonomic description of the species in line with the present-day requirements. A phylogenetic analysis based on 16S rRNA gene sequences and 120 conserved proteins from the 435^T^ genome revealed that this strain formed a separate clade within the genus *Desulfofundulus*. Overall genomic relatedness indices (OGRIs), such as ANI, AAI, and dDDH values, highlighted genomic differences between the new taxon and closely related type strains of *Desulfofundulus* species. Phenotypic, genomic, and phylogenomic data supported the classification of strains 435^T^ and 781 as members of a novel species with the proposed name *Desulfofundulus salinus* sp. nov., whose description is reported in Table 3. Comparative genomic analysis of strain 435^T^ unraveled the main aspects of the ecophysiology of bacteria inhabiting deep subsurface environments and increased the taxonomic diversity of the genus *Desulfofundulus*.

## Figures and Tables

**Table 1:** The similarity level (%) of the 16S rRNA gene sequences and genome relatedness indices (%) between strain 435^T^ and type strains of the *Desulfofundulus* species.

Type Strain	Genome	Genome Size, Mb	Genomic G + C Content, mol.%	Strain 435^T^		
				16S rRNA	dDDH	ANI
Strain 435^T^	GCF_003627965.1	2.88	55.1	100.0	100.0	100.0
*D. australicus* AB33^T^	GCF_900129285	2.88	54.6	93.6	27.0	83.0
*D. kuznetsovii* 17^T^	GCF_000214705	3.60	54.9	98.0	51.1	92.7
*D. luciae* SLT ^T^	GCF_030813795	2.95	55.2	94.7	52.2	93.3
*D. thermocisternus* ST90^T^	GCF_000686645	2.65	54.7	97.5	27.8	83.4
*D. thermosubterraneus* RL50JIII^T^	GCF_900142025	3.41	54.9	95.6	47.5	92.1
*D. solfataricus* V21^T^	ND *	ND	ND	98.4	ND	ND
*D. thermoacetoxidans* CAMZ^T^	ND	ND	ND	94.3	ND	ND
*D. thermobenzoicus* subsp. *thermobenzoicus* TSB^T^	ND	ND	ND	92.2	ND	ND
*D. thermobenzoicus* subsp. *thermosyntrophicus* TPO	GCF_009428905	3.71	55.8	95.2	25.0	78.7

* ND, no data.

**Table 2:** A comparison of the characteristics of strains 435^T^ and 781 with those of reference strains of the genus *Desulfofundulus*.

Characteristic	Strain, Species										
	1	2	3	4	5	6	7	8	9	10	11
Motility	+	+	+	+	+	+	–	+	+	+	+
Temperature range, °C	45–70	45–70	50–85	40–74	41–75	50–70	48–65	50–72	45–65	40–70	45–62
Temperature optimum, °C	55–60	55–60	60–65	68	62	60–65	60	61–66	55–60	62	55
NaCl range/optimum, % (*w*/*v*)	0–3/ 1	0–4.5/ 0.5	0–3/ 0	>0/ ND	0–4.7/ 0.3–1.2	0–3/ <1	6.4–7.9/7.3	0–1.5/ 0–1	0–1.5/ 0	0/ 0	ND
pH range	6.0–8.5	6.0–8.5	ND	5.5–8.5	6.2–8.9	6.3–8.3	0–1.5	6.4–7.8	6.0–7.5	6–8	6–8
pH optimum	7.0–8.0	7.0–8.0	7.0–7.2	7–7.4	6.7	7.0–7.9	0	7.2–7.4	6.5	7.2	7.0
Electron donors with sulfate:											
Formate	+	+	+	–	–	+	+	+	+	+	ND
Acetate	–	–	+	+	–	–	+	–	+	+	–
Propionate	+	+	+	–	+	–	+	+	+	+	+
Butyrate	+	+	+	–	+	–	+	+	+	+	–
Valerate	+	+	+		+	ND		+		+	ND
Hexadecanoate	+	+	+	+	+	ND	+	+	–	ND	ND
Fumarate	+	+	+	–		ND	+	+	ND	+	ND
Malate	+	+	+	–		–	+	+	+	+	–
Succinate	+	+	+	–		–	+	+	+	–	–
Benzoate	–	–	–	+	–	ND	–	–	–	+	+
Methanol	+	+	+	–	–	–	+	–	ND	–	–
Ethanol	+	+	+	+	+	+	+	+	–	+	–
Propanol	+	+	+	ND	+	ND	+	+	+	+	–
Butanol	+	+	+	ND	+	–	+	+	+	+	–
Glucose	–	–	–	–		–	+W	–	–	–	–
Fructose	–	–	–	ND	–	–	+	–	ND	–	–
L-Alanine	–	–	–	ND		ND	–	+	+	ND	ND
Electron acceptors:											
Thiosulfate	+	+	+	ND	+	+	+	+	+	+	–
Sulfite	+	+	+	ND	+	+–	+	+	–	+	–
Sulfur	–	–	–	ND	–	+	+	+	+	–	ND
Nitrate	–	–	–	ND	–	–	–	–	–	+	–
Lactate fermentation	–	–	+	+	ND	+	+	+	–	+	+

Strains: 1, 435^T^, this study and [8,9]; 2, 781, this study and [8]; 3, *D. kuznetsovii* 17^T^, this study and [14]; 4, *D. australicus* AB33^T^ [65]; 5, *D. thermocisternus* ST90^T^ [66]; 6, *D. luciae* SLT^T^ [67]; 7, *D. solfataricus* V21^T^ [68]; 8, *D. thermosubterraneus* RL50JIII^T^ [69]; 9, *D. thermoacetoxidans* CAMZ^T^ [70]; 10, *D. thermobenzoicus* subsp. *thermobenzoicus* TSB^T^ [71]; and 11, *D. thermobenzoicus* subsp. *thermosyntrophicus* TPO [72]. All taxa were characterized by rod-shaped cells, spore formation, fermentation of pyruvate, and utilization of lactate, pyruvate, and H_2_/CO_2_ as carbon and energy donors for sulfate-reduction. Designations: ‘+’, positive reaction; (W), weakly positive reaction; ‘−’, negative reaction; ND, not determined.

**Table 3:** Protologue description of *Desulfofundulus salinus* sp. nov.

Parameter	* Desulfofundulus salinus * sp. nov.
Genus name	* Desulfofundulus *
Species name	* Desulfofundulus salinus *
Species status	sp. nov.
Species etymology	sa.li’nus. N.L. masc. adj. *salinus*, salty
Designation of the Type Strain	435^T^
Strain Collection Numbers	VKM B-1492^T^ = DSM 23196^T^
Genome accession number	GCF_003627965.1
Genome status	Incomplete
Genome size	2886 kbp
GC mol%	55.1
16S rRNA gene accession nr.	AY918122
Description of the new taxon and diagnostic traits	The cells are rod-shaped or spindle-shaped (lemon-shaped), motile due to peritrichous flagella, stained Gram-negative, but have cell wall structure typical of Gram-positive bacteria. Endospores are spherical, located centrally or subterminally and slightly distend the cells. Growth is observed in the presence of 0–4.5% (*w*/*v*) NaCl (optimum, 0.5–1% NaCl), at a pH of 6.0–8.5 (optimum, pH 7.0), and at 45–70 °C (optimum, 55–60 °C) in a sulfate-reducing condition. Strictly anaerobic. Reduces sulfate to sulfide in media with H_2_/CO_2_, formate, lactate, pyruvate, malate, fumarate, succinate, methanol, ethanol, propanol, butanol, propionate, butyrate, valerate, and palmitate as carbon and energy sources, but does not use L-alanine, L-serine, L-arginine, L-cysteine, glucose, fructose, lactose, benzoate, citrate, tartrate, glycerol, glutamate, threonine, tryptophan, asparagine, glutamate, and phenylalanine. Lactate is oxidized with the production of acetate. No vitamins or other growth factors are required, although the addition of yeast extract stimulates growth. Pyruvate and (weakly) fumarate are used for fermentative growth, but lactate is not fermented. Utilizes sulfate, thiosulfate, and sulfite as electron acceptors in the presence of lactate, but does not use nitrate. The predominant cellular fatty acids are *iso*-C_15:0_, *iso*-C_17:0_, C_16:0_, and C_18:0_. The genome size of the type strain is 2.886 Mb with a genomic G + C content of 55.1 mol%. The type strain, 435^T^ (=VKM B-1492^T^ =DSM 23196^T^), was isolated from the Igrim gas field, in Khanty-Mansiysk Autonomous Okrug, Yugra, Russian Federation. The GenBank/EMBL/DDBJ accession number for the 16S rRNA gene sequence is AY918122 and the genomic assembly accession number is GCF_003627965.1. The reference strain is 781 (VKM B-1379).
Country and region of origin	Russian Federation, Khanty-Mansiysk Autonomous Okrug, Yugra
Date of isolation	1973
Source of isolation	A mixture of condensation and reservoir water from the Igrim gas field
Sampling date	1973
Latitude, Longitude	63°06′2.12″ N, 64°19′54.15″ E
Depth (meters below sea level)	1600–1620
Number of strains in study	2
Source of non-type strain	Strain 781 was isolated from the Ust-Balyk oil and gas field, Russia
Information related to the Nagoya Protocol	Not applicable

## Data Availability

The whole-genome shotgun project of strain 435^T^ has been deposited at DDBJ/EMBL/GenBank under the accession GCF_003627965.1, and it is the first version described in this paper.

## Supplementary Materials

The following supporting information can be downloaded at: https://www.mdpi.com/article/10.3390/microorganisms12061115/s1, Figure S1: Circular genome map of the *D. salinus* strain 435^T^. Abbreviations: *dsrAB*, dissimilatory sulfite reductase subunits A, B; *hydABC*, trimeric confurcating [FeFe]-hydrogenases; *fdhAB*, confurcating selenocysteine-incorporated formate dehydrogenase (NADP+); *phsA*, thiosulfate reductase/polysulfide reductase; *hndABCD*, tetrageteromeric NADP-dependent [Fe-Fe] hydrogenase; *foldD*, methenyltetrahydrofolate cyclohydrolase/methylenetetrahydrofolate dehydrogenase (NADP+); *metF, 1-2,* methylenetetrahydrofolate reductase; *acsA* (*cooS*), carbon monoxide dehydrogenase; *acsB*, acetyl-CoA synthase; *ascC*, acetyl-CoA synthase corrinoid iron–sulfur protein, large subunit; *ascD*, acetyl-CoA synthase corrinoid iron–sulfur protein, small subunit; *acsE*, methyltetrahydrofolate methyltransferase; *aprAB*, adenylylsulfate reductase subunit A, B; *spo0B*, sporulation response regulatory protein; *fhs*, formate-tetrahydrofolate ligase; *mtaA*, [methyl-Co(III) methanol-specific corrinoid protein]:coenzyme M methyltransferase; *mtaB*, methanol–corrinoid protein Co-methyltransferase; *mtaC*, methanol methyltransferase corrinoid protein; Figure S2: KEGG-map of carbon fixation pathways based on the genome analysis of the strain 435^T^. The enzymes annotated in the genome are highlighted in green. The Wood–Ljungdahl pathway is highlighted in pink; Figure S3: A cluster of genes presumably encoding enzymes of the carbonyl branch of the Wood–Ljungdahl pathway in the genome of the strain *D. salinus* 435^T^ compared to other bacteria. Abbreviations: *acsA (cooS)*, carbon monoxide dehydrogenase; *acsB*, acetyl-CoA synthase; *ascC*, acetyl-CoA synthase corrinoid iron–sulfur protein, large subunit; *ascD*, acetyl-CoA synthase corrinoid iron-sulfur protein, small subunit; *acsE*, methyltetrahydrofolate methyltransferase; *cooC*, carbon monoxide dehydrogenase maturation factor. Scale bar, 1000 bp; Figure S4: KEGG-map of sulfur metabolism pathways based on the genome analysis of strain 435^T^. The enzymes annotated in the genome are highlighted in green; Figure S5: KEGG-map of propanoate metabolism pathways based on the genome analysis of strain 435^T^. The enzymes annotated in the genome are highlighted in green; Figure S6: Gene organization of the mmc-cluster encoding propanoate oxidation in *D. salinus* 435^T^ and *D. kuznetsovii* 17^T^. Abbreviations: *mmcB*, fumarase, N-terminal domain; *mmcC*, fumarase, C-terminal domain; *mmcD1*, succinyl-CoA synthetase, beta subunit; *mmcD2*, succinyl-CoA synthetase, alpha subunit; *mmcE*, methylmalonyl-CoA mutase, N-terminal domain; *mmcG*, methylmalonyl-CoA epimerase; *mmcH*, methylmalonyl-CoA decarboxylase, alpha subunit; *mmcI*, methylmalonyl-CoA decarboxylase, epsilon subunit; *mmcJ*, methylmalonyl-CoA decarboxylase, gamma subunit; *mmcK*, malate dehydrogenase; *mmcL*, transcarboxylase 5S subunit. Scale bar, 1000 bp; Figure S7: Gene organization of the MT operon encoding a cobalt-dependent methyl transferase pathway in strains 435^T^ and *D. kuznetsovii* 17^T^. Abbreviations: 1, cobalamin-binding protein; *mtaA*, [methyl-Co(III) methanol-specific corrinoid protein]:coenzyme M methyltransferase; *mtaB*, methanol-corrinoid protein Co-methyltransferase; *mtaC*, methanol methyltransferase corrinoid protein; 2, cobalamin synthesis protein P47K; 3, ferredoxin iron–sulfur binding domain protein; 4, uroporphyrinogen decarboxylase; 5, methyltransferase cognate corrinoid protein; 6, ferredoxin; 7, tetrahydromethanopterin S-methyltransferase; 8, FMN-binding protein. Scale bar, 1000 bp; Figure S8: KEGG-map of methane metabolism pathways based on the genome analysis of strain 435^T^. The enzymes annotated in the genome are highlighted in green; Figure S9: KEGG-map of nitrogen metabolism pathways based on the genome analysis of strain 435^T^. The enzymes annotated in the genome are highlighted in green; Figure S10: Organization of gene clusters presumably encoding nitrogen fixation enzymes in the genome of strain 435^T^ and some other sulfate-reducing bacteria. Abbreviations: *nifH*, nitrogenase (molybdenum–iron) reductase and maturation protein, (*nifDK*), nitrogenase (molybdenum–iron) alpha and beta subunits; *nifB*, nitrogenase FeMo-cofactor synthesis FeS; nitrogen-fixation associated, P-II, nitrogen regulatory proteins; *nifN*, nitrogenase molybdenum–iron protein; *anfO*, Fe-only nitrogenase accessory AnfO family protein; *nrgA*, ammonium transporter; *nifV*, homocitrate synthase. Scale bar, 1000 bp; Figure S11: H_2_S production profiles of strains 435^T^ and 781 growing in fumarate–sulfate medium for 14 days at various temperatures (a) and NaCl concentrations (b); Table S1: Cellular fatty acid composition (%) of strains 435^T^, 781 and *D. kuznetsovii* 17^T^.
