# Supplementary material for: A Novel View on the Taxonomy of Sulfate-Reducing Bacterium ‘Desulfotomaculum salinum’ and a Description of a New Species Desulfofundulus salinus sp. nov
Source: Microorganisms. 2024 May 30;12(6):1115. doi: 10.3390/microorganisms12061115 (PMC11206085; doi:10.3390/microorganisms12061115)
Supplement: Supplementary file 1 [file microorganisms-12-01115-s001.zip › microorganisms-3026080-supplementary.pdf]

Supplementary Materials

A Novel View on the Taxonomy of Sulfate-Reducing Bacterium '*Desulfotomaculum salinum*' and a Description of a New Species *Desulfofundulus salinus* sp. nov.

Tamara N. Nazina [1,*], Tatyana P. Tourova [1], Denis S. Grouzdev [2], Salimat K. Bidzhieva [1] and Andrey B. Poltaraus [3]

[1] Winogradsky Institute of Microbiology, Research Center of Biotechnology, Russian Academy of Sciences, Moscow 119071, Russia; nazina@inmi.ru (T.N.N.); tptour@rambler.ru (T.P.T.); salima.bidjieva@gmail.com (S.K.B.)
[2] SciBear OU, Tartu mnt 67/1-13b, 10115 Tallinn, Estonia; denisgrouzdev@gmail.com (D.S.G.)
[3] Engelhardt Institute of Molecular Biology, Russian Academy of Sciences, 119991 Moscow, Russia; abpolt@gmail.com (A.B.P.)

* Correspondence: nazina@inmi.ru (T.N.N.); Tel.: +7-499-135-0341

This file includes:

Figures S1 to S11

Table S1

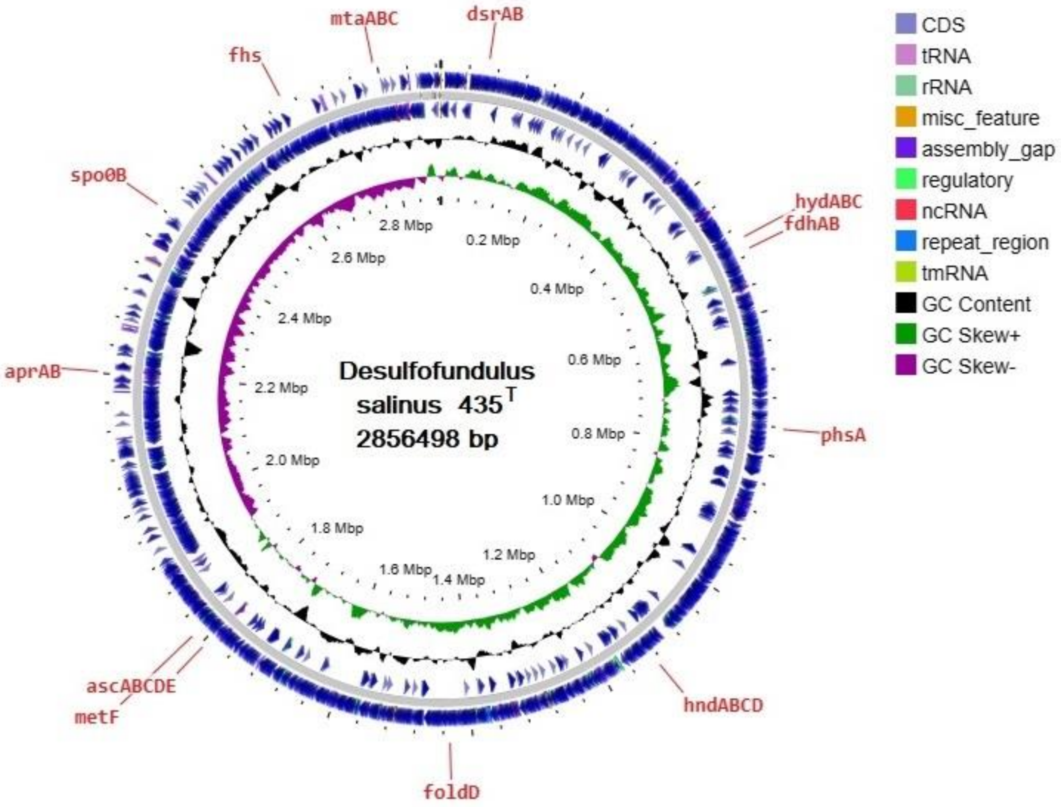

Figure S1. Circular genome map of the *D. salinus* strain 435^T. Abbreviations: *dsrAB*, dissimilatory sulfite reductase subunits A, B; *hydABC*, trimeric confurcating [FeFe]-hydrogenases; *fdhAB*, confurcating selenocysteine-incorporated formate dehydrogenase (NADP+); *phsA*, thiosulfate reductase / polysulfide reductase; *hndABCD*, tetrageteromeric NADP-dependent [Fe-Fe] hydrogenase; *foldD*, methenyltetrahydrofolate cyclohydrolase / methylenetetrahydrofolate dehydrogenase (NADP+); *metF, 1-2,* methylenetetrahydrofolate reductase; *acsA* (*cooS*), carbon monoxide dehydrogenase; *acsB*, acetyl-CoA synthase; *ascC*, acetyl-CoA synthase corrinoid iron-sulfur protein, large subunit; *ascD*, acetyl-CoA synthase corrinoid iron-sulfur protein, small subunit; *acsE*, methyltetrahydrofolate methyltransferase; *aprAB*, adenylylsulfate reductase subunit A, B; *spo0B*, sporulation response regulatory protein; *fhs*, formate--tetrahydrofolate ligase; *mtaA*, [methyl-Co(III) methanol-specific corrinoid protein]:coenzyme M methyltransferase; *mtaB*, methanol–corrinoid protein Co-methyltransferase; *mtaC*, methanol methyltransferase corrinoid protein.

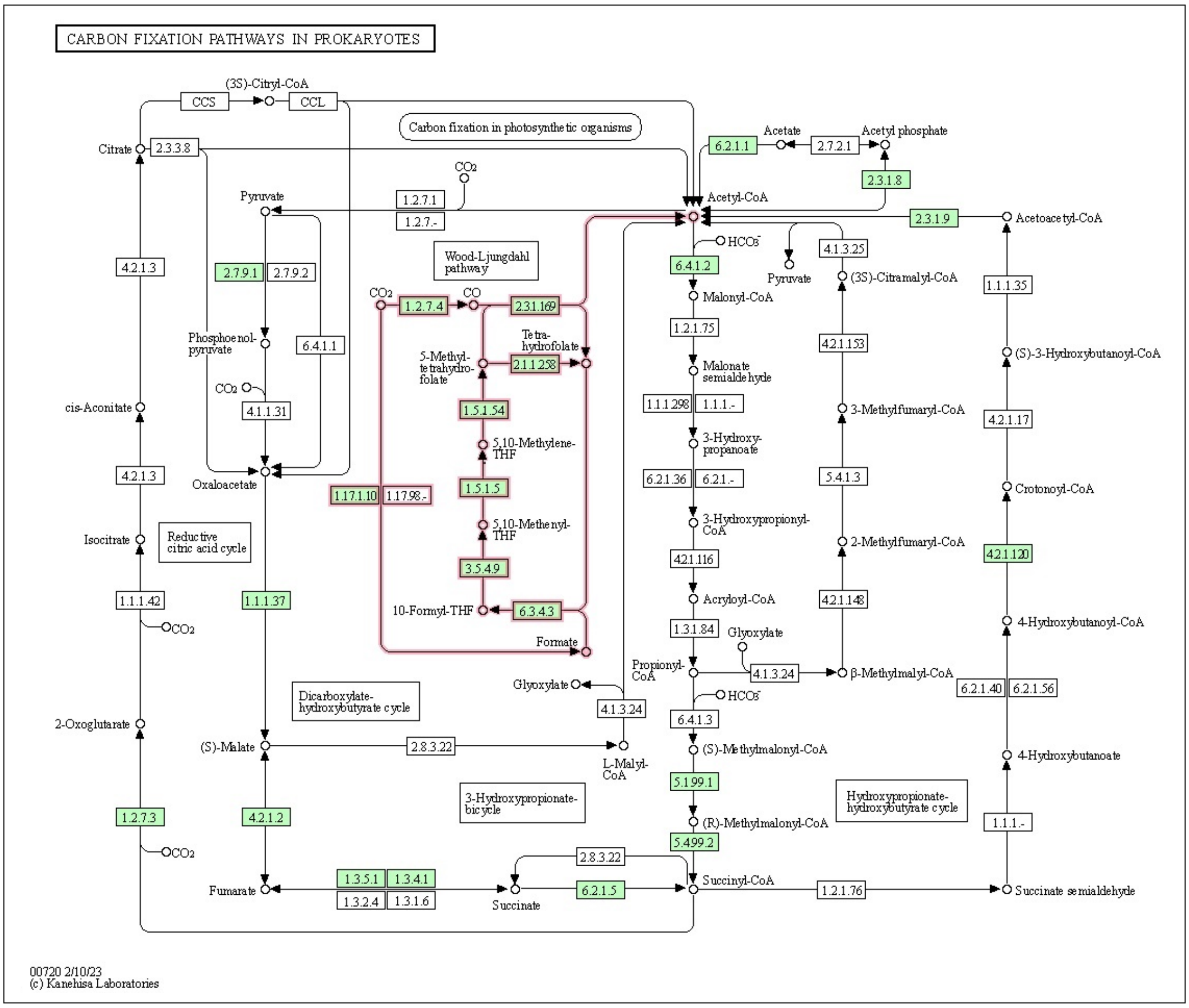

Figure S2. KEGG-map of carbon fixation pathways based on the genome analysis of the strain 435^T. The enzymes annotated in the genome are highlighted in green. The Wood-Ljungdahl pathway is highlighted in pink.

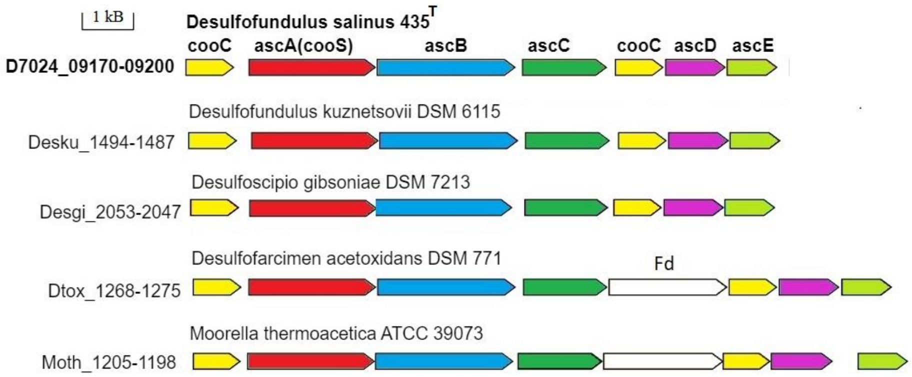

Figure S3. A cluster of genes presumably encoding enzymes of the carbonyl branch of the Wood-Ljungdahl pathway in the genome of the strain *D. salinus* 435^T compared to other bacteria. Abbreviations: *acsA (cooS)*, carbon monoxide dehydrogenase; *acsB*, acetyl-CoA synthase; *ascC*, acetyl-CoA synthase corrinoid iron-sulfur protein, large subunit; *ascD*, acetyl-CoA synthase corrinoid iron-sulfur protein, small subunit; *acsE*, methyltetrahydrofolate methyltransferase; *cooC*, carbon monoxide dehydrogenase maturation factor. Scale bar, 1000 bp.

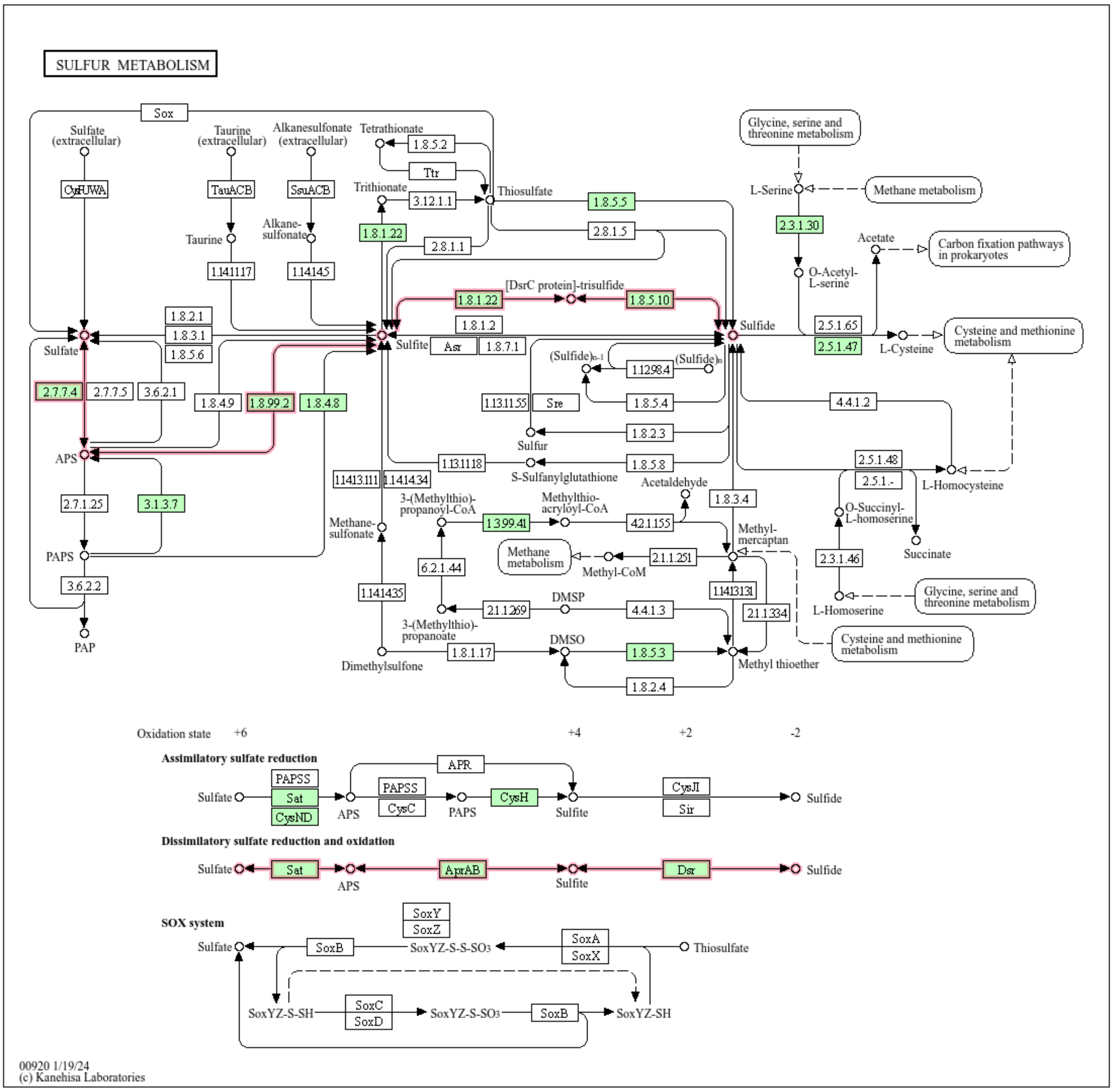

Figure S4. KEGG-map of sulfur metabolism pathways based on the genome analysis of strain 435[T]. The enzymes annotated in the genome are highlighted in green.

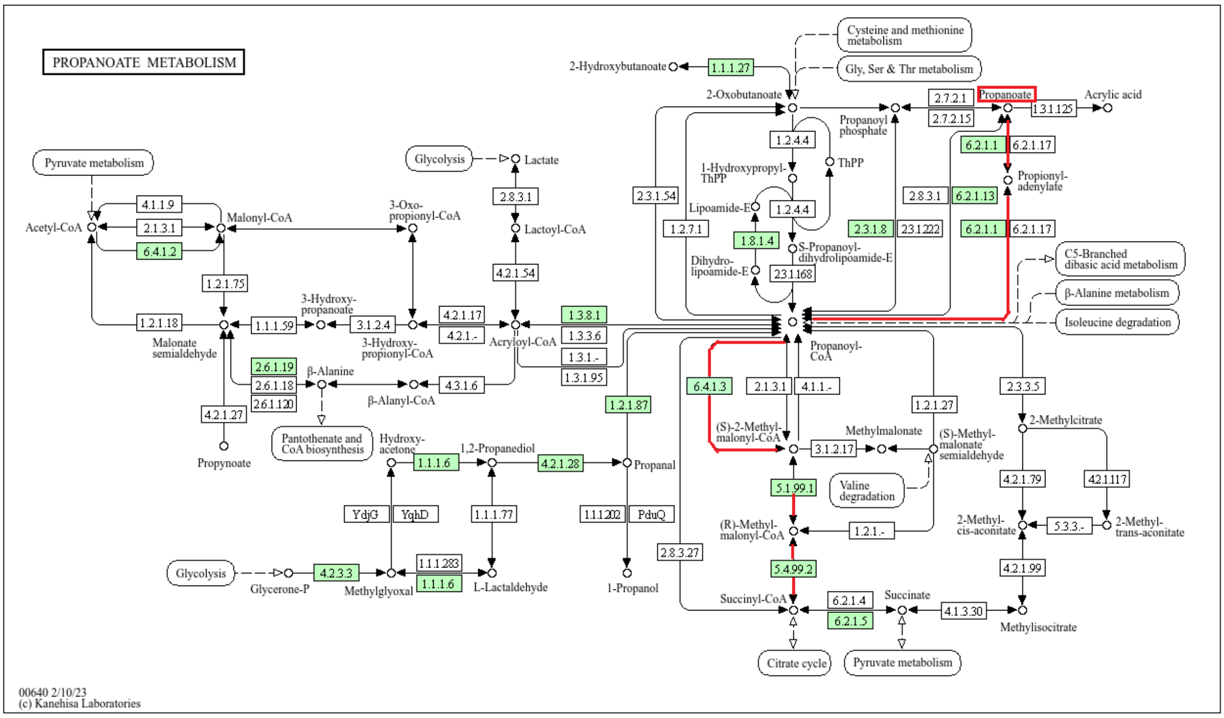

Figure S5. KEGG-map of propanoate metabolism pathways based on the genome analysis of the strain 435^{T}. The enzymes annotated in the genome are highlighted in green.

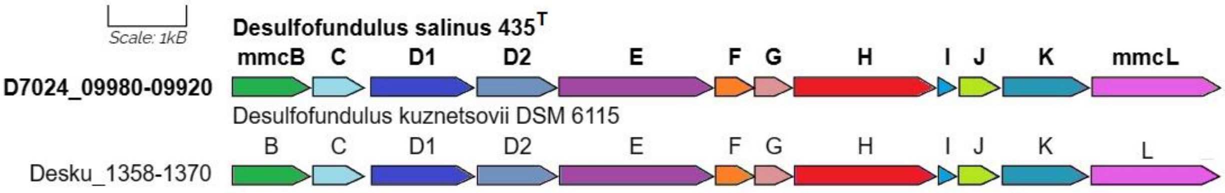

Figure S6. Gene organization of the mmc-cluster encoding propanoate oxidation in *D. salinus* 435^{T} and *D. kuznetsovii* 17^{T}. Abbreviations: *mmcB*, fumarase, N-terminal domain; *mmcC*, fumarase, C-terminal domain; *mmcD1*, succinyl-CoA synthetase, beta subunit; *mmcD2*, succinyl-CoA synthetase, alpha subunit; *mmcE*, methylmalonyl-CoA mutase, N-terminal domain; *mmcG*, methylmalonyl-CoA epimerase; *mmcH*, methylmalonyl-CoA decarboxylase, alpha subunit; *mmcI*, methylmalonyl-CoA decarboxylase, epsilon subunit; *mmcJ*, methylmalonyl-CoA decarboxylase, gamma subunit; *mmcK*, malate dehydrogenase; *mmcL*, transcarboxylase 5S subunit. Scale bar, 1000 bp.

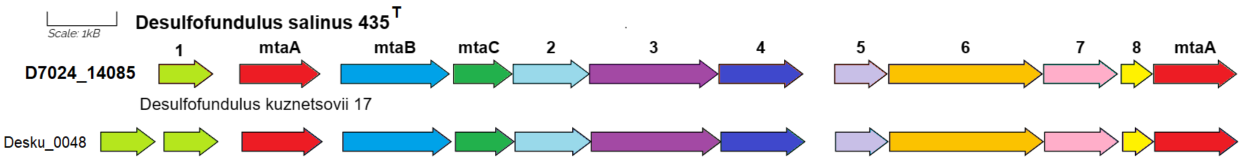

Figure S7. Gene organization of the MT operon encoding a cobalt-dependent methyl transferase pathway in strains 435^{T} and *D. kuznetsovii* 17^{T}. Abbreviations: 1, cobalamin-binding protein; *mtaA*, [methyl-Co(III) methanol-specific corrinoid protein]:coenzyme M methyltransferase; *mtaB*, methanol-corrinoid protein Co-methyltransferase; *mtaC*, methanol methyltransferase corrinoid protein; 2, cobalamin synthesis protein P47K; 3, ferredoxin iron-sulfur binding domain protein; 4, uroporphyrinogen decarboxylase; 5, methyltransferase cognate corrinoid protein; 6, ferredoxin; 7, tetrahydromethanopterin S-methyltransferase; 8, FMN-binding protein. Scale bar, 1000 bp.

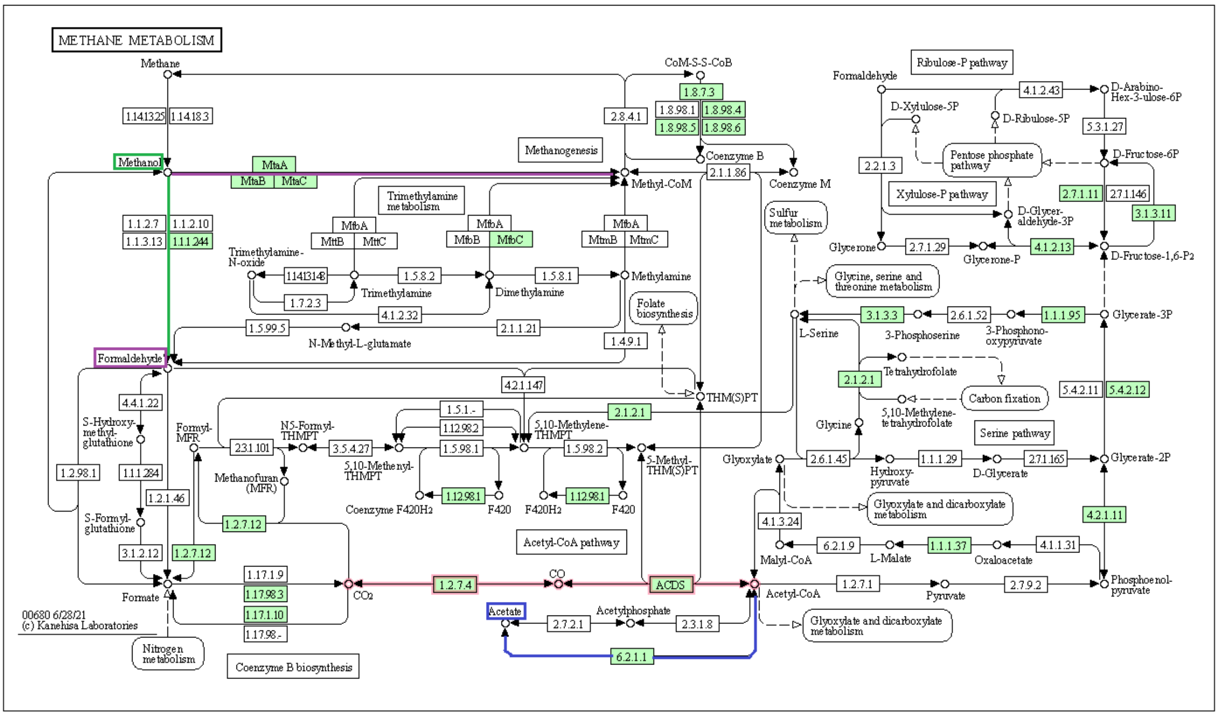

Figure S8. KEGG-map of methane metabolism pathways based on the genome analysis of strain 435^{T}. The enzymes annotated in the genome are highlighted in green.

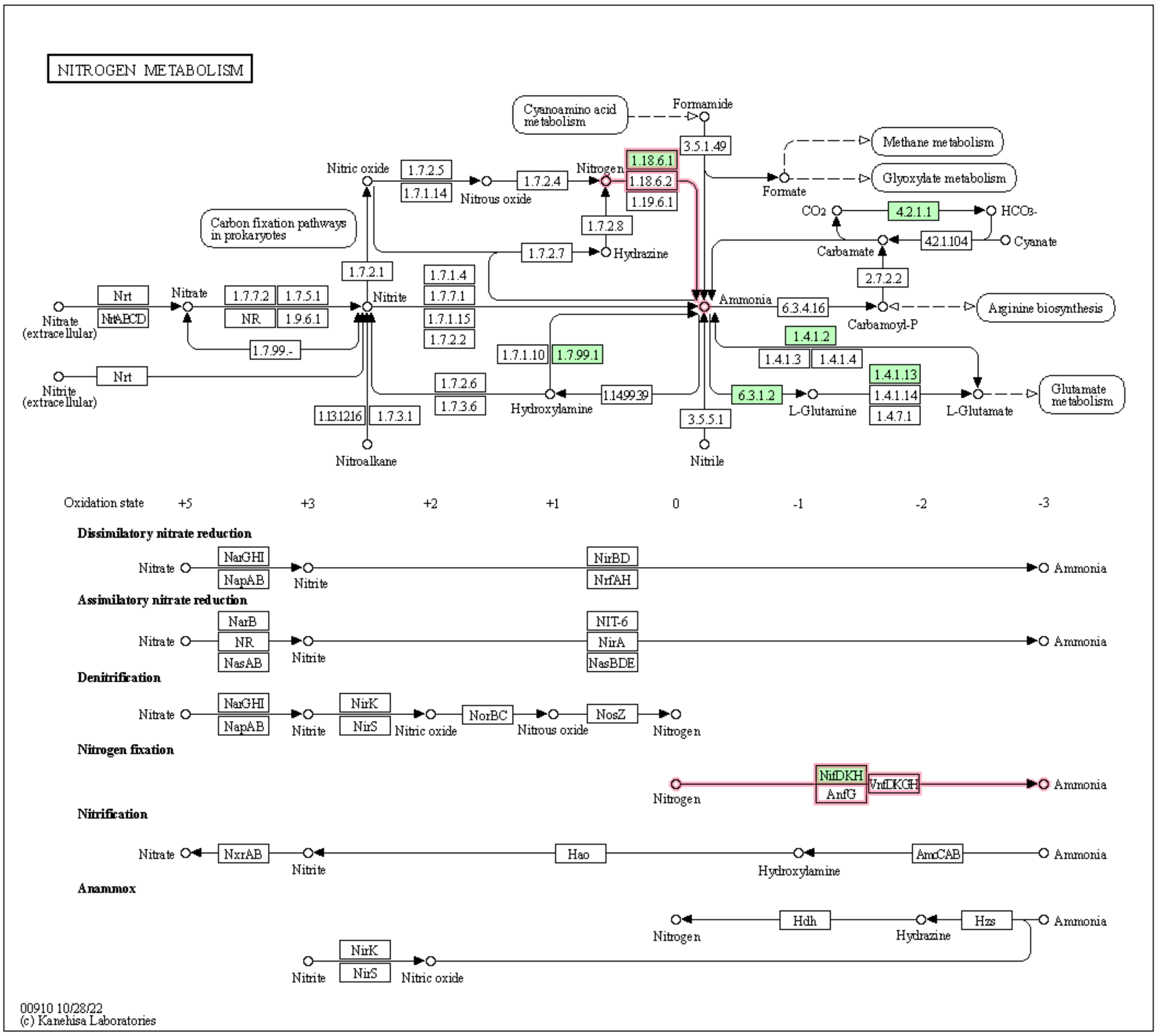

Figure S9. KEGG-map of nitrogen metabolism pathways based on the genome analysis of strain 435^{T}. The enzymes annotated in the genome are highlighted in green.

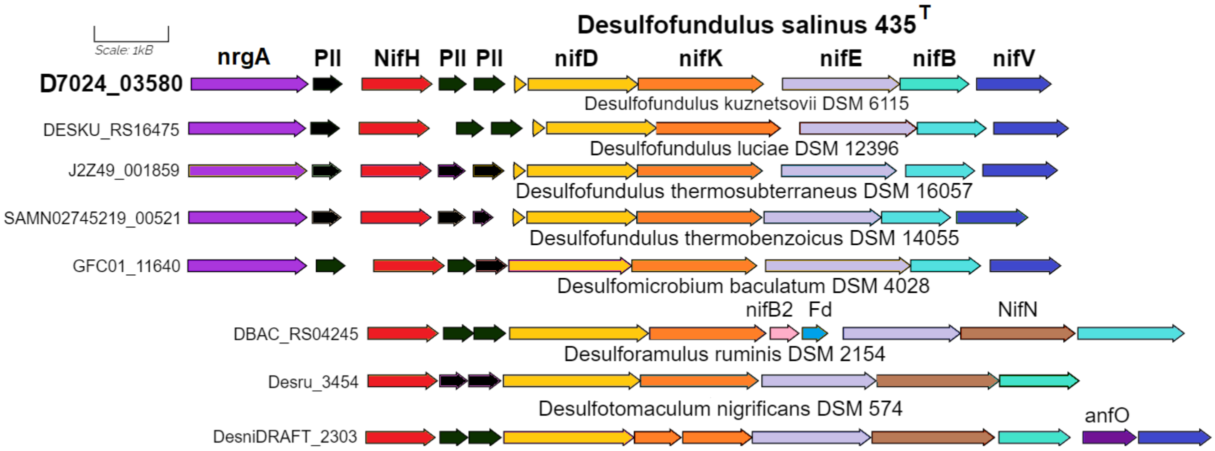

Figure S10. Organization of gene clusters presumably encoding nitrogen fixation enzymes in the genome of strain 435T and some other sulfate-reducing bacteria. Abbreviations: *nifH*, nitrogenase (molybdenum-iron) reductase and maturation protein, (*nifDK*), nitrogenase (molybdenum-iron) alpha and beta subunits; *nifB*, nitrogenase FeMo-cofactor synthesis FeS; nitrogen-fixation associated, P-II, nitrogen regulatory proteins; *nifN*, nitrogenase molybdenum-iron protein; *anfO*, Fe-only nitrogenase accessory AnfO family protein; *nrgA*, ammonium transporter; *nifV*, homocitrate synthase. Scale bar, 1000 bp.

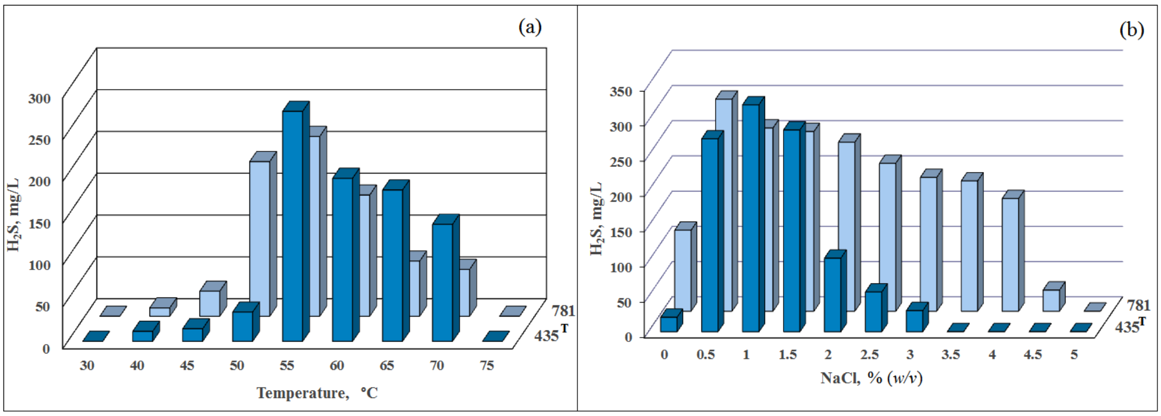

Figure S11. H_2S production profiles of strains 435T and 781 growing in fumarate-sulfate medium for 14 days at various temperatures (a) and NaCl concentrations (b).

Table S1. Cellular fatty acid composition (%) of strains 435[T], 781 and *D. kuznetsovii* 17[T].

Fatty acid	435[Ta]	781[a]	*D. kuznetsovii* 17[T]
$C_{14:0}$	2.2	3.2	3.3
***Iso*-$C_{15:0}$**	**22.1**	**32.7**	**29.8**
$C_{15:0}$	1.9	1.5	6.0
$C_{16:1}$	1.3	0.9	3.0
**$C_{16:0}$**	**21.1**	**25.5**	**22.4**
***Iso*-$C_{17:0}$**	**9.4**	**18.0**	**10.7**
$C_{17:0}$	0.8	0.8	1.9
$C_{18:1}$ *w9*	3.9.	4.9	
$C_{18:1}$ *w7*	1.2	0.7	
**$C_{18:0}$**	**31.5**	**10.7**	**20.3**
$C_{19:0}$	1.1	0.1	
$C_{20:0}$	2.1	0.3	
Others	1.4	0.7	2.6
Total	100.0	100.0	100.0

The values are percentages (*w*/*w*) of total fatty acids.
